# Adaptive Evolution of *Sphingobium hydrophobicum* C1^T^ in Electronic Waste Contaminated River Sediment

**DOI:** 10.3389/fmicb.2019.02263

**Published:** 2019-10-02

**Authors:** Da Song, Xingjuan Chen, Meiying Xu, Rong Hai, Aifen Zhou, Renmao Tian, Joy D. Van Nostrand, Megan L. Kempher, Jun Guo, Guoping Sun, Jizhong Zhou

**Affiliations:** ^1^School of Biology and Biological Engineering, South China University of Technology, Guangzhou, China; ^2^State Key Laboratory of Applied Microbiology Southern China, Guangdong Provincial Key Laboratory of Microbial Culture Collection and Application, Guangdong Institute of Microbiology, Guangdong Academy of Sciences, Guangzhou, China; ^3^Department of Plant Pathology and Microbiology, University of California, Riverside, Riverside, CA, United States; ^4^Institute for Environmental Genomics, Department of Microbiology and Plant Biology, University of Oklahoma, Norman, OK, United States

**Keywords:** *Sphingobium*, electronic waste (e-waste), xenobiotic degradation, heavy metal resistance, comparative genomics, genome plasticity, adaptive evolution

## Abstract

Electronic waste (e-waste) has caused a severe worldwide pollution problem. Despite increasing isolation of degradative microorganisms from e-waste contaminated environments, the mechanisms underlying their adaptive evolution in such habitats remain unclear. Sphingomonads generally have xenobiotic-degrading ability and may play important roles in bioremediation. *Sphingobium hydrophobicum* C1^T^, characterized with superior cell surface hydrophobicity, was recently isolated from e-waste contaminated river sediment. To dissect the mechanisms driving its adaptive evolution, we evaluated its stress resistance, sequenced its genome and performed comparative genomic analysis with 19 other *Sphingobium* strains. Strain C1^T^ can feed on several kinds of e-waste-derived xenobiotics, exhibits a great resistance to heavy metals and possesses a high colonization ability. It harbors abundant genes involved in environmental adaptation, some of which are intrinsic prior to experiencing e-waste contamination. The extensive genomic variations between strain C1^T^ and other *Sphingobium* strains, numerous C1^T^-unique genes, massive mobile elements and frequent genome rearrangements reflect a high genome plasticity. Positive selection, gene duplication, and especially horizontal gene transfer drive the adaptive evolution of strain C1^T^. Moreover, presence of type IV secretion systems may allow strain C1^T^ to be a source of beneficial genes for surrounding microorganisms. This study provides new insights into the adaptive evolution of sphingomonads, and potentially guides bioremediation strategies.

## Introduction

Electronic waste (e-waste) has become one of the most rapidly growing pollutants worldwide. It contains various toxic compositions, such as heavy metals, polychlorinated biphenyls (PCBs), polybrominated diphenyl ethers (PBDEs), phthalate esters (PAEs), and polycyclic aromatic hydrocarbons (PAHs). Severe contamination has been detected around e-waste disposal areas, especially in river sediment due to the strong adsorption of pollutants and slow natural attenuation (Leung et al., 2006; Zhang K. et al., 2012; Ma et al., 2013).

Environmental stress plays a critical role in the evolution of organisms, and extreme stress may lead to extinction, evolutionary changes and speciation (Nevo, 2011). The complex combined pollution resulting from e-waste has significantly altered soil and sediment microbiomes (Liu et al., 2015, 2018). Understanding the microbial adaptive evolution mechanisms for such complexly contaminated environments will contribute to modeling function-oriented evolution, obtaining beneficial biological parts and designing bioremediation strategies. Some microorganisms capable of degrading typical e-waste pollutants have been isolated from e-waste contaminated environments (Hu et al., 2015; Tang et al., 2016). However, little is known about their adaptive evolution processes.

Sphingomonads (*Sphingomonas* in a broader sense including *Sphingobium*) are often isolated from environments contaminated by organic pollutants and generally have the capability to degrade a variety of recalcitrant organic compounds, such as PAHs and halogenated aromatics, suggesting that they adapt well to contaminated environments and play important roles in bioremediation (Stolz, 2009). They mainly employ abundant oxygenases to degrade these xenobiotics (Stolz, 2009). They can synthesize carotenoids which protect cells from reactive oxygen species produced during degradation process (Liu et al., 2012). Their general physiological characteristics, including special outer membrane component glycosphingolipids, biosurfactant excretion, biofilm formation and chemotaxis, help them access to nutrients or pollutants (Cunliffe and Kertesz, 2006; Coppotelli et al., 2010). Comparative genomic analysis of sphingomonads have revealed diversity in the genomic organizations and genetic characteristics involved in pollutant degradation (Aylward et al., 2013; Verma et al., 2014; Wang et al., 2018), marine adaptation (Gan et al., 2013), nitrate respiration (García-Romero et al., 2016), etc. However, to the best of our knowledge, only two sphingomonads, *Sphingobium fuliginis* HC3 and *Sphingomonas* sp. MXB8, isolated from e-waste contaminated environments have been reported, and no genomic information is available. They are both from soil samples. Strain HC3 can degrade biphenyl and PCBs without dead-end intermediates accumulation (Hu et al., 2015), while strain MXB8 shows an excellent potential for the bioleaching of Ag from e-waste (Díaz-Martínez et al., 2019).

*Sphingobium hydrophobicum* C1^T^ was recently isolated from the sediment of the Lianjiang River in Guiyu, China, which had been contaminated for more than two decades by wastewater discharged from e-waste disposal (Chen et al., 2016). The sediment contains high concentration of heavy metals (Cu, 528 mg/kg; Zn, 249 mg/kg; Ni, 120 mg/kg; etc.), PAHs (3034 μg/kg), PBDEs (9054 μg/kg), PCBs (743 μg/kg) (Supplementary Table S1). Strain C1^T^ is the most hydrophobic sphingomonad ever known. Its high cell surface hydrophobicity (CSH), which may enhance colonization ability and adsorption of hydrophobic nutrients, is greatly attributed to the increased expression of certain outer membrane proteins (Chen et al., 2017). This study aims to address (i) what metabolic potential and genes enable strain C1^T^ to adapt to the stress from e-waste contaminated sediment? (ii) what are the main mechanisms driving its adaptive evolution? We evaluated its capability to cope with the main environmental stressors, sequenced its genome and performed comparative genomic analysis with 19 other *Sphingobium* strains isolated from different environments. The results demonstrated advantageous physiological characteristics, beneficial genetic elements and high genome plasticity of strain C1^T^, highlighted the important role of horizontal gene transfer (HGT) in its adaptive evolution, and suggested its role of beneficial-gene contributor.

## Materials and Methods

### Strains and Culture Conditions

*Sphingobium hydrophobicum* C1^T^ (= CCTCC AB 2015198 = KCTC 42740) was isolated from e-waste contaminated sediment in Guiyu, China (Chen et al., 2016). Strain C2, a hydrophilic variant of strain C1^T^, was obtained by cell passage for ∼100 generations in LB medium (Chen et al., 2017). Strains C3 and C4 were isolated from the first-round subculture (within 10 generations) of strains C1^T^ and C2 in LB medium, respectively. *Sphingobium xenophagum* NBRC 107872 was purchased from China General Microbiological Culture Collection Center. *E. coli* ATCC 25922 was deposited in our laboratory. *Sphingobium* strains were cultivated aerobically in LB medium or mineral salt medium (Chen et al., 2016) at 30°C. *E. coli* strain was cultivated aerobically in LB medium at 37°C.

### Xenobiotic Degradation Assay

Strain C1^T^ was cultivated in the mineral salt buffer with different xenobiotics (∼500 mg/L) as the sole carbon source. The tested xenobiotics included dimethyl phthalate, dibutyl phthalate, biphenyl, diphenyl ether, chlorobenzene, bromobenzene, etc. (Table 1). The growth of strain C1^T^, which was determined by naked-eye observation and protein quantification, indirectly reflected its degradation ability. Megascopic cell aggregates would appear and/or the medium would become turbid if strain C1^T^ could grow. The protein was quantified by the Coomassie brilliant blue method as described previously (Fang et al., 2015). All cultures were prepared in triplicate.

**TABLE 1:** The xenobiotic-degrading ability of strain C1^T^.

**Xenobiotics**	**Growth**
Dimethyl phthalate	+
Dibutyl phthalate	+
Biphenyl	+
*p*-Xylene	+
Ethylbenzene	+
Phenol	+
Diphenyl ether	+
Chlorobenzene	+
Bromobenzene	+
1,2-Dichlorobenzene	–
Phenylamine	–
Benzene	–
Phenanthrene	–
Pyrene	–
*n*-Hexadecane	–

*“+”, Megascopic cell aggregates appear and/or the medium becomes turbid within 7 days. “−”, no obvious growth within 7 days.*

### Heavy Metal Resistance Assay

The bacterial resistance to Cr(VI), Cu^2+^, Zn^2+^, Hg^2+^, Cd^2+^, Ni^2+^, and Pb^2+^ was determined by observing the growth in LB medium containing the corresponding compounds [K_2_Cr_2_O_7_, CuCl_2_, ZnCl_2_, HgCl_2_, CdCl_2_, NiCl_2_, Pb(NO_3_)_2_]. The number and size of bacterial colonies were recorded for solid culture, while the optical density of cultures at 600 nm was measured for liquid culture. All assays were performed in triplicate.

### CSH Assay

The CSH of cells in the late-exponential growth phase was measured using the microbial adhesion to hydrocarbon (MATH) protocol as described previously (Chen et al., 2017).

### Adhesion Assay

The bacterial adhesion capacity to kaolinite was evaluated as described previously (Yee et al., 2000) with slight modifications. Briefly, cells were harvested after overnight growth in LB medium and washed three times with 1 mM KNO_3_. Weighted cells were suspended in 30 mL of 1 mM KNO_3_, placed in contact with 4 g/L kaolinite, and equilibrated by shaking at 150 rpm for 1.5 h. Separation of kaolinite-adsorbed and free cells was performed by horizontal centrifugation (5000 rpm, 15 min) with 60 wt% sucrose. The free cells floating on top of the sucrose layer were harvested, measured using a spectrophotometer (600 nm), and then converted into weight. The adhesion intensity was determined by subtracting the weight of free cells from the initial weight. All assays were performed in triplicate.

### Biofilm Formation Assay

Biofilm formation ability was determined as described previously (Grzegorz et al., 2016) with slight modifications. Overnight cultures were diluted 100 times with LB medium and transferred into 96-well plates (150 μL/well). After 12, 24, 36, and 48 h static incubation, the biofilms in wells were washed three times with 0.9% NaCl and quantified by crystal violet staining. All assays were performed in sextuplicate.

### Replicon Detection

The replicons in strain C1^T^ and its variants (C2, C3, C4) were detected by in-gel cell lysis and pulse field gel electrophoresis. *Salmonella enterica* serovar Braenderup H9812 digested with *Xba*I was used as a size marker.

### Genome Sequencing

The genomes of strains C1^T^ and C2 were sequenced on PacBio RS II sequencing platform (Pacific Biosciences, United States).

### Data Analysis

#### Statistical Analysis

Multiple comparisons of means were performed using SPSS (v20.0) with one-way ANOVA. Bonferroni test was employed for equal variances assumed, while Tamhane’s T2 was employed for unequal variances.

#### Genome Assembly

*De novo* assembly of reads was performed by SMRT analysis pipeline v2.3.0 (Chin et al., 2013).

#### Gene Prediction and Functional Annotation

Open reading frames (ORFs) were identified by Prodigal (Hyatt et al., 2010). The rRNA, tRNA and other ncRNA were predicted using RNAmmer (Lagesen et al., 2007), ARAGORN (Laslett and Canback, 2004) and Infernal conjunction with Rfam (Nawrocki et al., 2009). The function of ORFs was annotated with EggNOG (Huertacepas et al., 2017), KEGG (Kanehisa et al., 2015), Swissprot and Nr. The β-barrel outer membrane proteins were predicted by PRED-TMBB2^1^ (Tsirigos et al., 2016). Two-component regulatory systems were predicted by P2RP^2^ (Barakat et al., 2013).

#### Phylogenetic Analyses

The phylogenetic tree based on 16S rRNA gene sequences was constructed using Mega 6.0 with ClustalW alignment method and Neighbor Joining algorithm (Tamura et al., 2013). Another phylogenetic tree based on shared gene families was constructed by CMG-biotools (Vesth et al., 2013). The average nucleotide identity (ANI) values were calculated using the JSpeciesWS server^3^ (Richter et al., 2016).

#### Prediction of Mobile Genetic Elements

Prophages were predicted by PHASTER^4^ (Arndt et al., 2016). Genomic islands (GIs) were predicted by IslandViewer4^5^ using the method SIGI-HMM and IslandPath-DIMOB both based on sequence composition (Bertelli, 2017). Insertion sequences (ISs) were detected by ISsaga^6^ (Varani et al., 2011). All the analyses were performed with default parameters.

#### Genomic Comparison Approaches

The C1^T^ genome was compared with the 19 *Sphingobium* genomes available (accession numbers in Supplementary Table S2) in the NCBI Genome Database. The core- and pan-genome analysis of these *Sphingobium* strains was performed using CMG-biotools with a threshold of 50% identity and 50% coverage for protein sequences (Vesth et al., 2013). The comparisons of genome/gene sequences were performed using BRIG (Alikhan et al., 2011), Mauve (Darling et al., 2004), Mummer (Kurtz et al., 2004), and MCScanX (Wang et al., 2012).

#### Test for Positive Selection

The identification of orthologs between strain C1^T^ and *S. xenophagum* strains was based on two-way best Blastp match (coverage ≥ 70%, identity ≥ 80%). Orthologs were further aligned by ParaAT2.0 (Zhang Z. et al., 2012). Then Ka/Ks (non-synonymous to synonymous substitution rate ratio) analysis for orthologs was performed using KaKs_Calculator Toolbox 2.0 with the maximum-likelihood method GY (Wang et al., 2010). The gene was judged to be under positive selection if Ka/Ks > 1 and *P*-value (Fisher) < 0.05.

## Results

### Stress Resistance of Strain C1^T^

For microbial survival and growth, the main stressors from e-waste contaminated river sediment may include toxic xenobiotics, heavy metals, current scour and frequent environmental fluctuations. Therefore, strain C1^T^ was expected to degrade several kinds of e-waste-derived xenobiotics, exhibit a great resistance to heavy metals and possess a high colonization ability.

First, xenobiotic-degrading ability of strain C1^T^ was tested, and result showed that strain C1^T^ could utilize various common xenobiotics as the sole carbon source for growth, such as PAEs, biphenyl, diphenyl ether, bromobenzene and chlorobenzene (Table 1 and Supplementary Figure S1).

**FIGURE 1:**
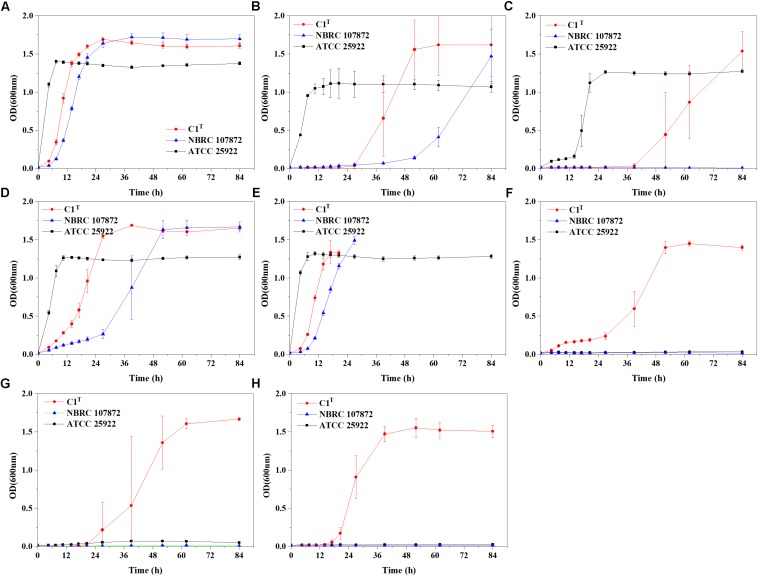
Growth curves of strains C1^T^, NBRC 107872 and ATCC 25922 in LB liquid medium with heavy metals. **(A)** Control group, with no heavy metal. **(B)** 3 mM Cu^2+^. **(C)** 0.3 mM Cr(VI). **(D)** 1.5 mM Ni^2+^. **(E)** 3 mM Pb^2+^. The cultures of strains C1^T^ and NBRC 107872 gradually became brownish black after OD_600_ exceeded 1.5. **(F)** 1 mM Cd^2+^. **(G)** 0.25 mM Hg^2+^. **(H)** 6 mM Zn^2+^. The values are means of triplicate investigations, and error bars indicate standard deviations. The error bars are large in **(B,C,G)** due to obvious differences in lag phases among three replicates of strain C1^T^.

Heavy metal resistance of strain C1^T^, *S. xenophagum* NBRC 107872 and *E. coli* ATCC 25922 was evaluated and compared. Strain NBRC 107872 is one of the most closely related strains. Strain ATCC 25922, a well-characterized Gram-negative strain, is widely used as a control for various laboratory experiments. As shown in Figure 1, strain C1^T^ could grow in LB medium containing Cu^2+^ (3 mM), Cr(VI) (0.3 mM), Ni^2+^ (1.5 mM), Pb^2+^ (3 mM), Cd^2+^ (1 mM), Hg^2+^ (0.25 mM) and Zn^2+^ (6 mM), respectively. It was more resistant to most of the tested heavy metals than strain NBRC 107872, and had a greater tolerance to Zn^2+^, Cd^2+^, and Hg^2+^ than strain ATCC 25922 (Figure 1 and Table 2).

**TABLE 2:** Heavy metal resistance of strains C1^T^, NBRC 107872 and ATCC25922.

	**Concentration (mM)**	**C1^T^**	**NBRC 107872**	**ATCC 25922**
Cr(VI)	0.2	±	–	±
	0.3	±/–	–	±/–
	0.4	–	–	–
Cu^2+^	2	+	+	+
	3	±/–	±	±/–
	4	–	–	–
Zn^2+^	2	+	–	–
	4	±/–	–	–
	6	±/–	–	–
Hg^2+^	0.1	+	–	–
	0.25	±	–	–
	0.5	–	–	–
Cd^2+^	0.5	±	–	±/–
	1	±	–	–
	2	–	–	–
Ni^2+^	1	+	+	+
	1.5	±	±/–	+
	2	–	–	±
Pb^2+^	2	+	+	+
	3	+	+	+
	4	–	–	±

*“+”, the growth rate and number of bacterial colonies are similar to those in control group (growth in LB solid medium without heavy metals); “±”, slower growth; “±/–”, slower growth and fewer bacterial colonies; “–”, no bacterial colonies.*

**FIGURE 2:**
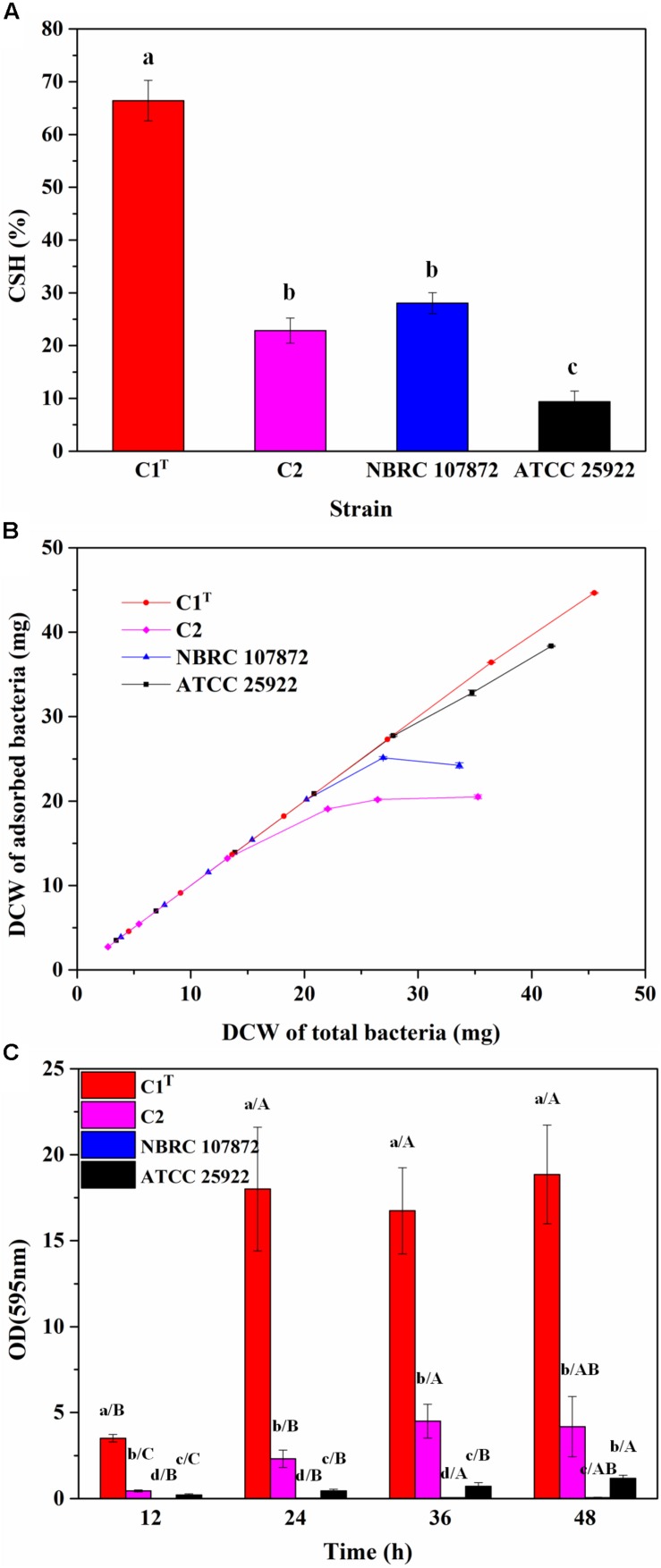
The colonization ability of strains C1^T^, C2, NBRC 107872 and ATCC 25922. **(A)** Cell surface hydrophobicity (CSH) measured by MATH protocol. The values are means of triplicate investigations, and error bars indicate standard deviations. Significant differences were tested by one-way ANOVA with Bonferroni test (*n* = 3, α = 0.05). The a, b and c represent the differences among these strains. **(B)** The adhesion capacity to kaolinite. Experiments were conducted with a fixed amount of kaolinite (4 g/L) and varying amounts of bacteria in 30 mL of 1 mM KNO_3_. The values are means of triplicate investigations, and error bars indicate standard deviations. DCW, (dry cell weight. **(C)** The biofilm-forming ability quantified by crystal violet staining. The values are means of sextuplicate investigations, and error bars indicate standard deviations. Significant differences were tested by one-way ANOVA with Tamhane’s T2 test (*n* = 6, α = 0.05). Lowercase letters represent the differences among these strains at the same incubation time, while capital letters represent the differences among different incubation time of a strain.)

In order to measure colonization ability of strain C1^T^, the CSH, adhesion capacity and biofilm-forming ability of strains C1^T^, C2, NBRC 107872 and ATCC 25922 were tested and compared. As shown in Figure 2, strain C1^T^ displayed the highest CSH, strongest adhesion capacity to kaolinite (a main soil/sediment component) and highest biofilm-forming ability, suggesting that it might be able to colonize efficiently in sediment.

### General Features of the C1^T^ Genome

The complete C1^T^ genome is 4.6 Mb and consists of two chromosomes and five large plasmids. General features are summarized in Table 3. The two largest replicons harbor 1 or 2 copies of rRNA gene operons, so are defined as chromosomes. The existence of a plasmid replication initiator in Chromosome 2 and its megaplasmid-like size suggest that it may originate from a plasmid followed by the uptake of some essential genes including rRNA genes. In total, 4506 ORFs are predicted, approximately 30% of which encode hypothetical proteins.

**TABLE 3:** General features of the C1^T^ genome.

	**Size (Kb)**	**GC%**	**ORFs**	**rRNA**	**tRNA**	**tmRNA**	**miscRNA**
Chromosome 1	3059.0	63.6	2979	3	52	1	6
Chromosome 2	711.6	61.96	638	6	6	0	2
Plasmid 1	270.5	63.89	278	0	0	0	1
Plasmid 2	247.5	62.5	265	0	0	0	0
Plasmid 3	171.6	62.37	185	0	0	0	1
Plasmid 4	79.9	62.91	99	0	0	0	0
Plasmid 5	62.6	59.26	62	0	0	0	0
Total	4602.6	63.19	4506	9	58	1	10

### Genomic Differences Between Strain C1^T^ and Other *Sphingobium* Strains

In order to find the genomic characteristics of strain C1^T^ involved in adaptive evolution, the C1^T^ genome was analyzed and compared with the genomes of 19 other *Sphingobium* strains isolated from different habitats (Supplementary Table S2). Based on 16S rRNA genes, shared gene families and ANI values, strain C1^T^ is most closely related to *S. xenophagum* QYY and NBRC 107872 (Supplementary Figure S2 and Supplementary Table S3). Strains C1, QYY and NBRC 107872 seem to belong to the same species since ANI values among them are more than 95% and their 16S rRNA genes are identical.

**FIGURE 3:**
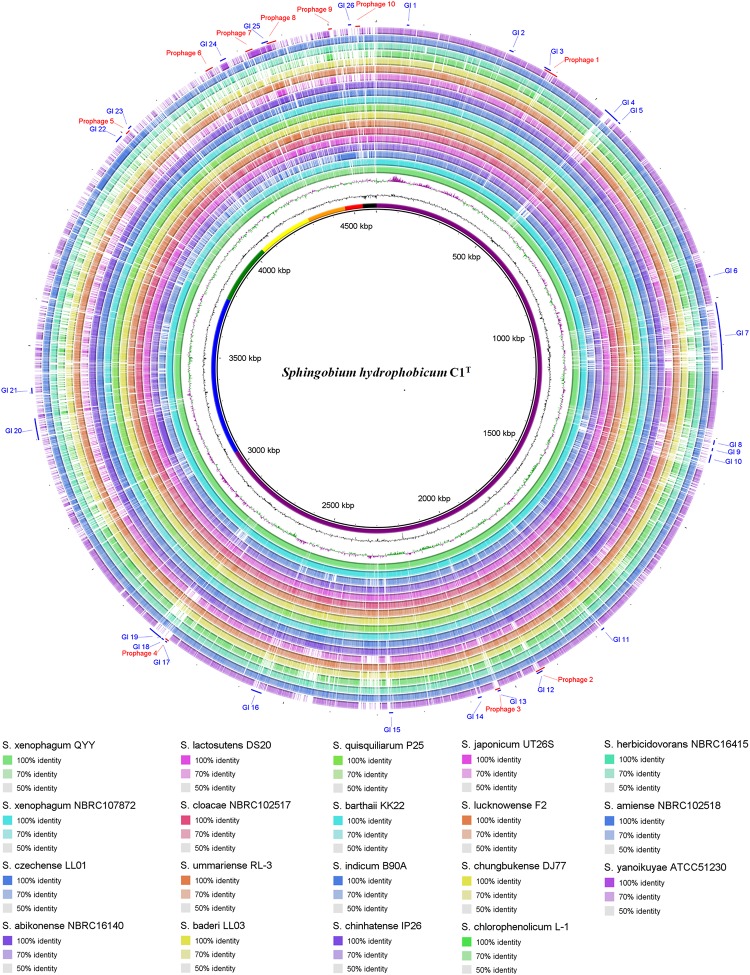
Circular representation of the C1^T^ genome and comparison results with other *Sphingobium* genomes. From inner to outer ring: (1) Seven replicons of strain C1^T^, (2) GC content, (3) GC skew, (4–21) Genome comparison (Blastn) results between strain C1^T^ and other *Sphingobium* strains listed at the bottom, (22) Prophages predicted by PHASTER, (23) GIs predicted by IslandViewer4.

Genome comparison between strain C1^T^ and other *Sphingobium* strains at the nucleotide level is shown in Figure 3. The main regions of Chromosome 1 are conserved among these strains, while Chromosome 2 and the plasmids vary greatly. Some gaps exactly match the predicted GIs and prophages. There are totally 26 GIs and 10 prophages in the C1^T^ genome.

**FIGURE 4:**
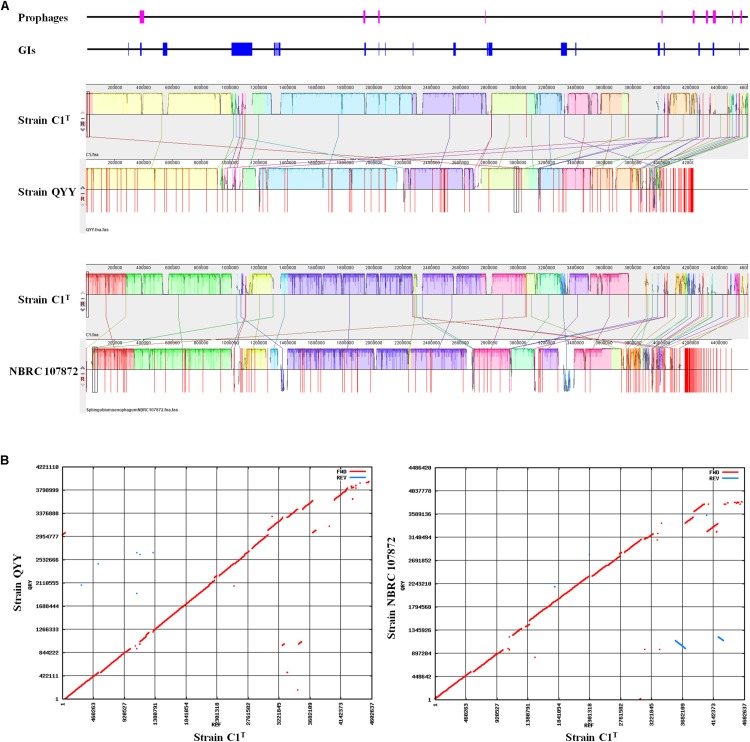
Genome alignment of strains C1^T^, QYY and NBRC 107872. **(A)** Genome alignment by Mauve. Each of colored blocks represents a presumably homologous region. Blocks above/below the center line respectively indicate aligned regions in the forward/reverse (reverse complement) orientation. The height of the similarity profile inside each block corresponds to the level of sequence conservation. The positions of prophages and GIs in the C1^T^ genome are shown correspondingly at the top. **(B)** Genome alignment by Mummer. Red/blue dots correspond to pairs of homologous sequences in the forward/reverse (reverse complement) orientation.

The C1^T^ genome was further aligned with the genomes of stains QYY and NBRC 107872 by Mauve and MUMmer. As shown in Figure 4, the order and direction of most regions are the same, and the gaps mainly correspond to plasmids, GIs and prophages. Genome alignments of strain C1^T^ and other *Sphingobium* strains show more genome rearrangements and gaps (Supplementary Figure S3).

### Core- and Pan-Genome of *Sphingobium* Strains

To uncover the genomic differences at ORF level, the core- and pan-genome analysis was conducted (Supplementary Figure S4). The core genome comprises 948 gene families, while the pan genome comprises 18036, suggesting a high genetic variation and great adaptive potential of *Sphingobium* strains.

In the C1^T^ genome, 1052 ORFs belong to the core genome, accounting for 23.3% of the total ORFs. Assuming they were randomly distributed on chromosomes and plasmids, 66.1% of them would be on Chromosome 1 (number of ORFs on Chromosome 1/total number of ORFs in the genome). In fact, 92.3% are on Chromosome 1, indicating its predominant importance in core physiological processes. Strain C1^T^ has 458 unique gene families consisting of 515 ORFs, approximately 50% of which are located on Chromosome 2 and the plasmids. A majority (70.3%) of these unique genes have no EggNOG annotation or belong to “Function unknown (S),” suggesting that strain C1^T^ has plenty of unique and unknown metabolic potential.

### Rapid Changes of the C1^T^ Genome in Laboratory Cultures

**FIGURE 5:**
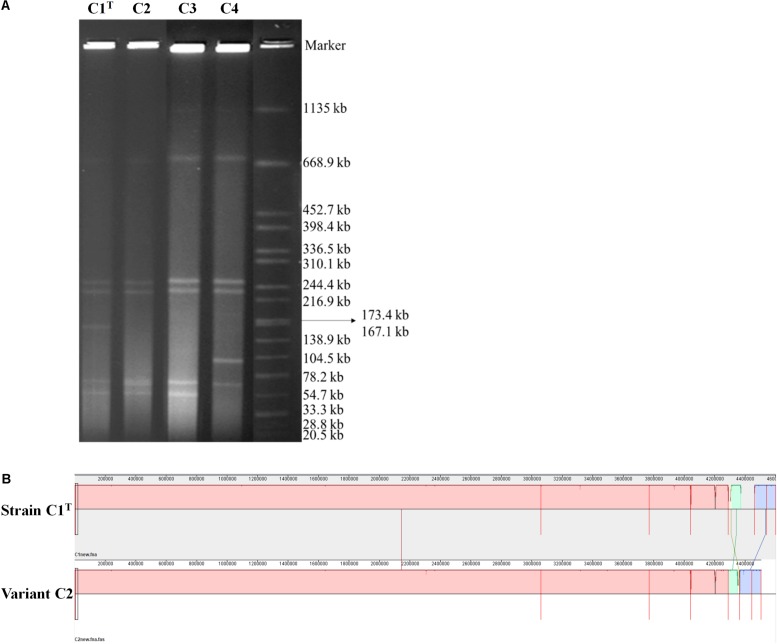
The genomic differences between strain C1^T^ and its variants. **(A)** The genome profiles detected by PFGE. *Salmonella enterica* serovar Braenderup H9812 digested with *Xba*I was used as a size marker. **(B)** Genome alignment of strains C1^T^ and C2 by Mauve.

Consistent with the high genome plasticity demonstrated by comparative genomic analysis, frequent genome rearrangements were observed in isolates from laboratory cultures. Variants C3 and C4 were isolated after one subculture of strain C1^T^ and hydrophilic variant C2 in LB medium, respectively. The profiles of replicons in these strains varied significantly (Figure 5A). Whole genome sequencing of variant C2 showed that an approximately 100 kb fragment on Plasmid 3 was missing (Figure 5B). This fragment is present on both Plasmids 1 and 3 of strain C1^T^ (Supplementary Figure S5) and is flanked by ISs, indicating the important role of ISs in genome plasticity. The C1^T^ genome harbors 91 ISs in total. In addition, 12 SNPs and 67 gaps between the C1^T^ and C2 genomes were identified.

### Mechanisms Underlying the Adaptive Evolution of Strains C1^T^

Positive selection, gene duplication (GD) and HGT are the common mechanisms driving microbial adaptive evolution. First, Ka/Ks analysis was employed to evaluate the selection pressure on orthologs. A majority of the orthologs between strains C1^T^ and NBRC 107872 are found to be under negative selection. However, approximately 40% of the orthologs (except those having no variation) between strains C1^T^ and QYY may be subjected to positive selection (Supplementary Table S4). These orthologs mainly belong to “Inorganic ion transport and metabolism (P),” “Cell wall/membrane/envelope biogenesis (M)” and “Energy production and conversion (C)” (Supplementary Figure S6).

Many homologous regions/genes among the chromosomes and plasmids of strain C1^T^ were observed (Supplementary Figure S5). Several regions belong to prophages which have similar DNA sequences. Others may be derived from GD and genome rearrangements, some of which contain ISs.

The sequences of 5 plasmids, 26 GIs and 10 prophages, which are related to HGT, account for 27.9% of the C1^T^ genome and contribute to the main genomic differences from closely related strains (Figures 3, 4). They contain many genes involved in environmental adaptation, e.g., GI 7 contains genes involved in type IV secretory system and heavy metal resistance; GI 21 encodes a complete peptide/nickel transport system and a haloacid dehalogenase. Interestingly, the plasmid sequences are found to have high similarity with plasmids of distant sphingomonads, and even different regions in a plasmid of strain C1^T^ are similar to plasmids from different sources (Supplementary Figure S7). For instance, the middle part of Plasmid 4 is similar to Plasmid unnamed1 of *Sphingomonas* sp. NIC1, and the both sides are similar to Plasmid of *Sphingopyxis* sp. 113P3. It suggests that plasmid transfer and rearrangement happen among sphingomonads. Additionally, the C1^T^ genome harbors 5 gene clusters encoding three categories of type IV secretion systems, 1 *trb*, 2 *tra* and 2 *vir*, which are responsible for conjugation process of plasmids belonging to different incompatibility groups.

The genes and metabolic potential involved in coping with the main stressors were further analyzed.

#### Xenobiotic Degradation

**FIGURE 6:**
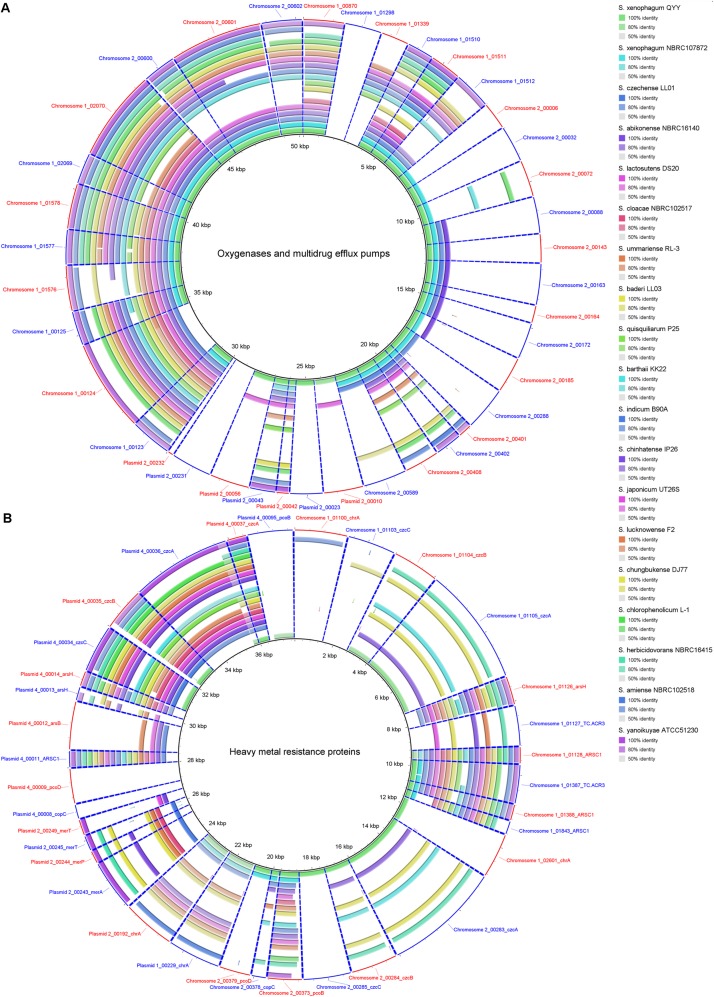
Homology search of related protein sequences of strain C1^T^ against the 19 other *Sphingobium* genomes. These proteins are listed in Supplementary Table S5. Each ring represents the Blastx comparison result against a genome. The presence/absence of homologous gene is visually presented, and the gradation of color indicates sequence identity. **(A)** Oxygenases and multidrug efflux pumps. **(B)** Heavy metal resistance proteins.

A total of 8 monooxygenase genes and 19 dioxygenase genes responsible for cleaving the ring of aromatic/cyclic compounds were annotated in the C1^T^ genome (Supplementary Table S5). They are scattered on Chromosome 1 (5), Chromosome 2 (15) and Plasmid 2 (7). Most have homologous sequences with high identity in strains QYY and NBRC 107872 (Figure 6A). Notably, the genes encoding alpha and beta subunits of biphenyl dioxygenase on Plasmid 2 are not present in the 19 other *Sphingobium* genomes. Instead, they are closely related to those of strains from *Actinobacteria* and next to an IS, suggesting that they were acquired via cross-phylum HGT. Two oxygenase genes (homogentisate 1,2-dioxygenase and camphor 5-monooxygenase) are found to be under positive selection (Supplementary Table S5).

The C1^T^ genome harbors complete degradation gene clusters for catechol, protocatechuate and PAEs. The catechol degradation gene cluster is located on GI 10 flanked by ISs, without homologous sequences in strains NBRC 107872 and QYY, suggesting that this gene cluster was acquired via HGT. In contrast, the protocatechuate degradation gene cluster on Chromosome 2 has homologous sequences in strains NBRC 107872 and QYY with almost identical genome positions, suggesting that this gene cluster existed before these strains diverged.

**FIGURE 7:**
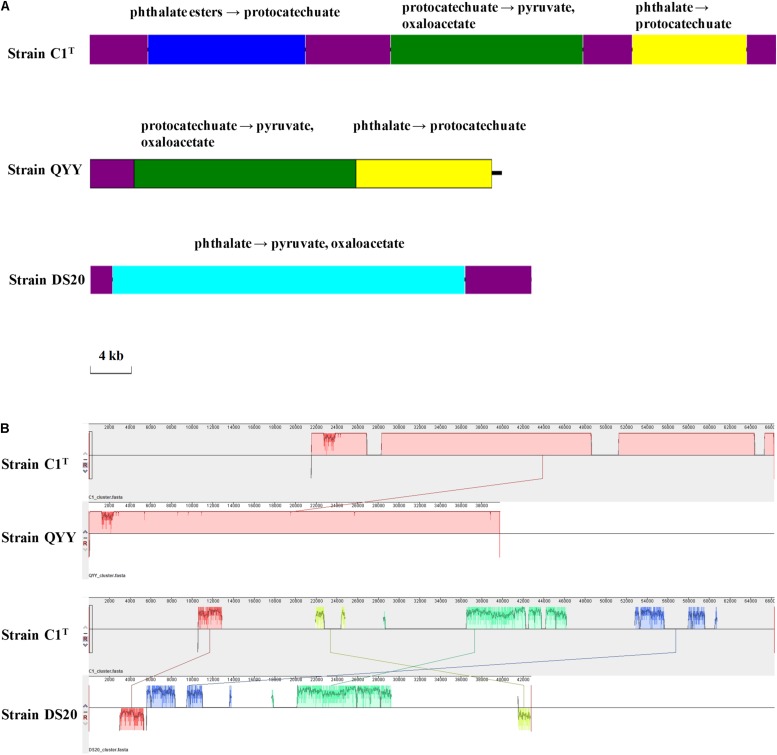
The PAEs degradation gene clusters of strains C1^T^, QYY and DS20. **(A)** Organizations of these gene clusters. The purple blocks represent ISs. Other blocks contain genes responsible for corresponding metabolic processes. **(B)** Homologous regions between these gene clusters detected by Mauve. The image scales of **(A,B)** are the same.

The PAEs degradation gene cluster on Plasmid 2 comprises three regions separated by ISs (Figure 7). The first and third regions contain homologous genes for transforming phthalate to protocatechuate, suggesting a GD event. The second region contains genes for degrading protocatechuate. Among the 19 other *Sphingobium* strains, only strains QYY and DS20 have homologous genes for transforming phthalate to protocatechuate. However, the genetic organizations are different among the three strains (Figure 7). These results suggest that the gene cluster may be derived from HGT initially and then undergo multiple translocations.

In addition, strain C1^T^ has 4 sets of multidrug efflux pump genes, 3 *acrAB* and 1 *emrAB* (Supplementary Table S5), which may confer resistance to toxic xenobiotics. One *acrAB* is located on Chromosome 2, and the other three sets on Chromosome 1. Most *Sphingobium* strains have homologous genes, suggesting that these genes are intrinsic.

#### Heavy Metal Resistance

Strain C1^T^ harbors 34 resistance genes for various heavy metals, such as chromate, copper, and mercury (Supplementary Table S5). Based on function annotation, toxic heavy metals could be either transformed into less toxic forms, or pumped out via efflux transporters. Among these genes, 11 are on Chromosome 1 (7 on GI 7), 6 on Chromosome 2, 5 on Plasmid 2 and 11 on Plasmid 4. Some resistance genes are multiple, e.g., 4 chromate transporter genes (*chrA*), 3 cobalt-zinc-cadmium efflux gene clusters (*czcABC*) and 2 copper resistance gene clusters. The two *chrA* genes on Plasmid 1 and Plasmid 2 have identical sequences, and are flanked by similar sequences, suggesting a large-fragment GD event. However, other genes/gene clusters share low to median amino acid identity (<80%), and their locations in the genome have no correlation, indicating that they may not have directly originated from GD events.

The resistance genes on chromosomes (except those on GI 7) are closely related to strain QYY, while the others are closely related to distant sphingomonads. Especially the resistance genes on Plasmid 4 are closely related to *Sphingopyxis* sp. 113P3. Many resistance genes of strain C1^T^ only have homologs in some *Sphingobium* strains which are not always close relatives (Figure 6B). Notably, closely related strain NBRC 107872 possesses few homologs. These indicate that heavy metal resistance genes could spread among sphingomonads via HGT, and that resistance genes in the C1^T^ genome may mainly originate from other sphingomonads by HGT. In addition, Ka/Ks analysis indicates that three resistance genes (*ARSC1*, *czcB* and *pcoD*) may be subjected to positive selection (Supplementary Table S5).

#### Cell Surface Hydrophobicity (CSH)

A total of 180 β-barrel outer membrane proteins were predicted in strain C1^T^, and they were compared against the 19 other *Sphingobium* genomes to identify candidates related to the high CSH (Supplementary Figure S8). Some only exist in strain C1^T^ and/or certain strains, suggesting that these strains may harbor unique transport channels and receptors. Strain NBRC 107872, isolated from a water sample of the Elbe River, has very similar β-barrel outer membrane proteins as strain C1^T^, but shows hydrophilic feature (Figure 2). The result suggests that the high CSH of strain C1^T^ is likely due to the abundance of some outer membrane proteins, and that the evolution of CSH appears to be affected by the habitat.

In addition, the genes involved in polysaccharide synthesis and secretion, which may affect CSH and colonization ability, are mainly located on Chromosome 1 and Plasmid 5. Notably, 29% of the ORFs on Plasmid 5 are assigned to “Cell wall/membrane/envelope biogenesis (M)” and primarily participate in polysaccharide synthesis and secretion. Among them, 10 are C1^T^-unique. The average GC content of Plasmid 5 is much lower than that of the whole genome, further suggesting that Plasmid 5 was acquired via HGT.

#### Two-Component Regulatory Systems

A total of 42 histidine kinases (7 are possible incomplete), 41 response regulators and 4 phosphotransfer proteins are predicted in the C1^T^ genome, and approximately 75% of them are located on Chromosome 1. The gene number is comparable to other *Sphingobium* strains, but is more than that of typical *E. coli* strains (the P2CS database). These genes include 11 known two-component regulatory systems involved in acidity sensing, nitrogen regulation, redox response, etc. (Supplementary Table S6). Most are also present in the 19 other *Sphingobium* genomes.

Notably, the DctB-DctD system, which senses the important carbon and energy source C4-dicarboxylates and regulates their transporter gene *dctA*, is only present in strains C1^T^, NBRC 16415 and ATCC 51230. However, the genes *dctB and dctD* of these strains are not homologous, and their organizations are different (Supplementary Figure S9). The FixL-FixJ system, which constitutes an oxygen-sensitive switch for regulating genes involved in nitrogen fixation and/or microaerobic respiration, has two gene copies on Plasmids 1 and 3, respectively. No homolog is found in the 18 other *Sphingobium* genomes (except NBRC 16415). These results indicate that the two-component regulatory systems DctB-DctD and FixJ-FixL may be acquired via HGT.

## Discussion

Microorganisms survive and thrive in various natural or artificial environments and play important roles in geochemical cycling and environmental remediation. Sphingomonads are often studied due to their metabolic versatility, especially xenobiotic-degrading ability, but these studies often overlook other phenotypic and genetic characteristics involved in adaptation to contaminated environments (Stolz, 2009; Aylward et al., 2013; Tabata et al., 2016). In this study, we focused on the important physiological characteristics of *S. hydrophobicum* C1^T^, a sphingomonad type strain isolated from e-waste contaminated river sediment, and employed comparative genomic analysis to uncover its adaptive evolution mechanisms.

**FIGURE 8:**
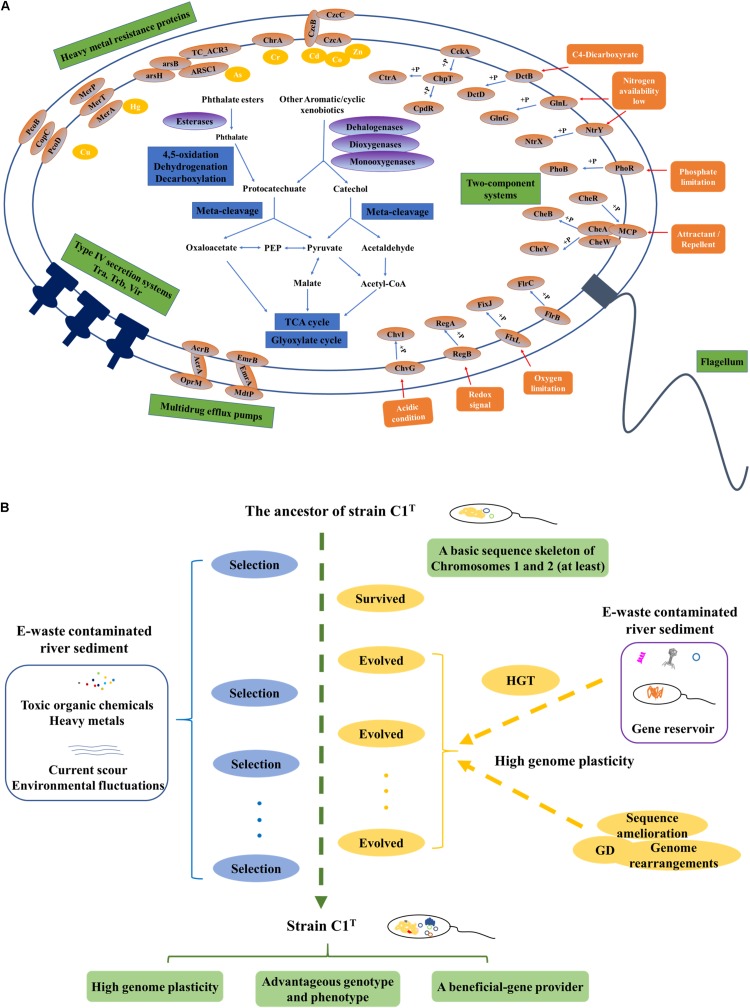
The adaptive evolution of strain C1^T^ in e-waste contaminated river sediment. **(A)** A brief metabolic reconstruction of strain C1^T^. The physiological processes and proteins involved in xenobiotic degradation, heavy metal resistance, environmental response and conjugational transfer are shown. **(B)** A conceptual model of the adaptive evolution process. Positive selection, gene duplication (GD), and especially horizontal gene transfer (HGT) drive the adaptive evolution of strain C1^T^. After rounds of selection, strain C1^T^ obtained a high genome plasticity and advantageous phenotype.

The first question addressed is “what metabolic potential and genes enable strain C1^T^ to adapt to the environmental stress.” The stressors from e-waste contaminated river sediment generally include a variety of toxic xenobiotics and heavy metals, current scour and frequent environmental fluctuations. Strain C1^T^ could feed on several kinds of e-waste-derived xenobiotics, exhibited a great resistance to some heavy metals, and possessed a high colonization ability. As other sphingomonads, various oxygenase genes are responsible for its xenobiotic degradation potential (Aylward et al., 2013). The specific biphenyl dioxygenase genes and PAEs degradation genes are involved in the degradation of e-waste-derived xenobiotics (Leung et al., 2006; Ma et al., 2013). Catechol and protocatechuate degradation gene clusters provide common downstream degradation pathways for aromatic compounds (Fuchs et al., 2011). Its heavy metal resistance is mainly attributed to multiple efflux pump genes, such as cobalt-zinc-cadmium efflux gene clusters (*czcABC*). Moreover, Hg^2+^ and As(III) can be transformed to less toxic forms by MerA and ArsH, respectively (Silver, 1996; Yang and Rosen, 2016). The genes for exopolysaccharide synthesis may contribute to colonization ability, heavy metal resistance and removal (Periasamy et al., 2015; Gupta and Diwan, 2017). Abundant two-component regulatory systems allow it to respond to frequent environmental fluctuations. Among them, specific system FixL-FixJ may help it adapt to oxygen-deficient condition of sediment (Rodgers and Lukat-Rodgers, 2005). In brief, its genome harbors abundant genes involved in coping with the stressors (Figure 8A).

The second question is “what are the main mechanisms driving the adaptive evolution of strain C1^T^.” Various microbial evolutionary mechanisms, including positive selection, GD and HGT, can result in a diverse array of metabolic potential, which in turn contributes to the rapid adaptation of microorganisms to environmental stress (Petersen et al., 2007; Andersson and Hughes, 2009; Hemme et al., 2016). However, the relative importance of these processes remains elusive. The genes involved in direct dynamic interactions with the environment are likely under positive selection (Petersen et al., 2007). GD occurs much more frequently than spontaneous point mutation (Andersson and Hughes, 2009). HGT may account for 1.6 ∼32.6% of the genes in each individual genome (Koonin et al., 2001). Previous comparison of 26 sphingomonad genomes revealed that selfish genetic elements might be prominent forces shaping genomes, and that megaplasmids, prophages, transposons, and frequent genome rearrangements were prevalent features in this group (Aylward et al., 2013). Multiple degradation genes are usually scattered in the genome and flanked by insertion elements, which may allow quicker adaptation to xenobiotics than other bacteria by forming new composite degradation pathways (Stolz, 2009). In this study, explicit evidence is provided for high plasticity of the C1^T^ genome, i.e., extensive genomic variations between strain C1^T^ and other *Sphingobium* strains, numerous C1^T^-unique genes, massive mobile elements and frequent genome rearrangements. Numerous genes involved in coping with the stressors originate from HGT, especially HGT among sphingomonads, e.g., the biphenyl dioxygenase genes on Plasmid 2 and the heavy metal resistance genes on GI 7 and plasmid 4. Some genes under positive selection and GD events are detected, but a few of them are directly involved in xenobiotic degradation and heavy metal resistance. Therefore, HGT may be dominant during the evolution of xenobiotic degradation potential and heavy metal resistance.

In addition, genome comparison between strain C1^T^ and its two most closely related strains QYY and NBRC 107872 provides clues about the origins of some genome elements. The history of e-waste recycling in the sediment sampling site Guiyu can be traced back to the late 1980s with a large increase in 1995 (Leung et al., 2006). Strains C1^T^, NBRC 107872 and QYY were isolated from different environments (Supplementary Table S2) before/in 2014 (Chen et al., 2016), 1986 (Nörtemann et al., 1986), and 2005 (Qu et al., 2006) respectively. Therefore, the genes of strain C1^T^, which have orthologs in strain NBRC 107872, are speculated to be intrinsic genes appearing prior to the e-waste contamination. These genes include the protocatechuate degradation gene cluster on Chromosome 2, multidrug efflux pump genes, most of the genes encoding two-component regulatory systems, etc. Likewise, the genes having orthologs in strain QYY can be speculated to appear before/during the early phase of e-waste contamination, e.g., some heavy metal resistance genes and part of the PAEs degradation gene cluster.

Based on these results, a possible conceptual model is proposed to describe the adaptive evolution of strain C1^T^ (Figure 8B). Prior to exposure to e-waste contamination, the ancestor of strain C1^T^ had at least a basic sequence skeleton of Chromosomes 1 and 2, likely harboring many oxygenase genes, the protocatechuate degradation gene cluster and a few heavy metal resistance genes. When initially exposed to e-waste contamination, i.e., an initial round of selection, it survived due to its natural resistance. The e-waste contaminated river sediment likely served as a “reservoir” of beneficial genes (Liu et al., 2018). Therefore, it had plenty of opportunities to acquire foreign plasmids and DNA fragments (mainly from sphingomonads) containing beneficial genes by transformation, conjugation and transduction. These plasmids and DNA fragments could exchange genes with the host genome by recombination, and/or be duplicated and inherited along the lineage. The high genome plasticity of strain C1^T^ strongly suggested the possibility of these processes. As a result, intrinsic genes and acquired foreign genes could collectively form new composite metabolic characteristics. The accumulation of mobile elements including plasmids, prophages and ISs further increased genome plasticity. In addition, GD, genome rearrangements and sequence amelioration might occur. After subsequent rounds of selection, evolved strain C1^T^ obtained a high genome plasticity and advantageous phenotype. The well-adapted strain C1^T^ may now serve as a beneficial-gene contributor to promote the adaptive evolution of surrounding microorganisms and consequently accelerate the bioremediation process.

Future studies on experimental evolution of sphingomonads or communities and the relationship between genotype and phenotype will further illuminate the adaptive evolution mechanisms of sphingomonads for complex e-waste contamination.

## Data Availability Statement

The C1^T^ genome sequences have been deposited in GenBank under the accession numbers CP022745, CP022746, CP022747, CP022748, CP022749, CP022750, and CP022751.

## Author Contributions

All authors conceived the study and revised the manuscript. DS, XC, MX, and RH designed the experiments. DS performed the experiments. DS, MX, AZ, and RT analyzed the data. DS wrote the manuscript.

## Conflict of Interest

The authors declare that the research was conducted in the absence of any commercial or financial relationships that could be construed as a potential conflict of interest.

## Footnotes

1 http://www.compgen.org/tools/PRED-TMBB2

2 http://www.p2rp.org/

3 http://jspecies.ribohost.com/jspeciesws/#Home

4 http://phaster.ca/

5 http://www.pathogenomics.sfu.ca/islandviewer/browse/

6 https://www-is.biotoul.fr
