# Supplementary material for: Adaptive Evolution of Sphingobium hydrophobicum C1T in Electronic Waste Contaminated River Sediment
Source: Front Microbiol. 2019 Oct 2;10:2263. doi: 10.3389/fmicb.2019.02263 (PMC6783567; doi:10.3389/fmicb.2019.02263)
Supplement: Supplementary file 1 [file Data_Sheet_1.zip › Data Sheet 1/Supplementary Materials/Supplementary materials_figure legends.docx]

**Supplementary materials**

**Figure legends**

**Figure S1.** The growth of strain C1^T^ in the mineral salt buffer with xenobiotics. (A) The representative photograph of cultures in the seventh day. Megascopic cell aggregates appear and/or the medium becomes turbid. (B) Protein contents of cultures in the seventh day. CK, the growth in the mineral buffer with no carbon source. The values are means of triplicate investigations, and error bars indicate standard deviations.

**Figure S2.** Phylogenetic analyses of strain C1^T^. (A) The phylogenetic tree based on 16S rRNA gene sequences. It was constructed using Mega 6.0 with ClustalW alignment method and Neighbor Joining algorithm (bootstrap value =1000). (B) The phylogenetic tree based on shared gene families. It was constructed by CMG-biotools.

**Figure S3.** Genome alignment of strains C1^T^ and other *Sphingobium* strains (*S. abikonense* NBRC 16140, *S. lactosutens* DS20 and *S. czechense* LL01). Each of colored blocks represents a presumably homologous region. Blocks above / below the center line respectively indicate aligned regions in the forward / reverse (reverse complement) orientation. The height of the similarity profile inside each block corresponds to the level of sequence conservation.

**Figure S4.** Core- and pan-genome plot of *Sphingobium* genus. The red and blue lines reflect the changes in protein family number of the core- and pan-genome, respectively. The black and gray bars indicate the number of new proteins and new protein families with the increase of *Sphingobium* genomes.

**Figure S5.** Homologous regions among the replicons of strain C1^T^. (A) Sequence alignment of the replicons by Mauve. (B) Gene mappings among the replicons. The lines connect homologous genes with ≥50% identity and ≥50% coverage.

**Figure S6.** COG category of the orthologs between strains C1^T^ and QYY. “C”, energy production and conversion; “D”, cell cycle control, cell division, chromosome partitioning; “E”, amino acid transport and metabolism; “F”, nucleotide transport and metabolism; “G”, carbohydrate transport and metabolism; “H”, coenzyme transport and metabolism; “I”, lipid transport and metabolism; “J”, translation, ribosomal structure and biogenesis; “K”, transcription; “L”, replication, recombination and repair; “M”, cell wall/membrane/envelope biogenesis; “N”, cell motility; “O”, posttranslational modification, protein turnover, chaperones; “P”, inorganic ion transport and metabolism; “Q”, secondary metabolites biosynthesis, transport and catabolism; “S”, function unknown; “T”, signal transduction mechanisms; “U”, intracellular trafficking, secretion, and vesicular transport; “V”, defense mechanisms; “NA”, no annotation.

**Figure S7.** Sequence alignments of the C1^T^ plasmids and highly similar plasmids. The species names in parentheses represent plasmid sources. Plasmid 5 has no similar sequence with high coverage in the NCBI Nucleotide Database.

**Figure S8.** Differences between strain C1^T^ and each *Sphingobium* strain in the beta-barrel outer membrane proteins. Each ring represents the Blastx comparison result with a representative genome (reference: the amino acid sequences of the beta-barrel outer membrane proteins in strain C1^T^).

**Figure S9.** Genetic organizations of C4-dicarboxylate transporter system in the genomes of strains C1^T^, ATCC 51230 and NBRC 16415. Each of colored blocks represents the gene shown above.
