# Supplementary material for: Adaptive Evolution of Sphingobium hydrophobicum C1T in Electronic Waste Contaminated River Sediment
Source: Front Microbiol. 2019 Oct 2;10:2263. doi: 10.3389/fmicb.2019.02263 (PMC6783567; doi:10.3389/fmicb.2019.02263)
Supplement: Supplementary file 1 [file Data_Sheet_1.zip › Data Sheet 1/Supplementary Materials/Table S1.docx]

**Table S1.** Concentrations of pollutants in sediments of the Lianjiang River in Guiyu

| Pollutants | Concentration | | |
| --- | --- | --- | --- |
| Heavy metals | mg/kg | | |
| Cd | 0.9^a^ | 0.556 ± 0.089^b^ | 14.081 ± 1.22^c^ |
| Cu | 528^a^ | 620.9 ± 478.9^b^ | 2977.432 ± 358.63^c^ |
| Ni | 120^a^ | 69.7 ± 68.7^b^ | n.a.^c^ |
| Pb | 94.3^a^ | 93.0 ± 52.4^b^ | 385.150 ± 43.12^c^ |
| Zn | 249^a^ | n.a.^b^ | 744.691 ± 36.54^c^ |
| Cr | 29.2^a^ | n.a.^b^ | 40.931 ± 7.93^c^ |
| Hg | n.a.^a^ | 0.493 ± 0.197^b^ | n.a.^c^ |
| As | n.a.^a^ | 11.3 ± 3.90^b^ | n.a.^c^ |
| PAHs | μg/kg | | |
| Naphthalene | 25.8^a^ | 159^d^ | 37.2±12.9^e^ |
| Acenaphthylene | n.d.^a^ | 36^d^ | n.d.^e^ |
| Acenaphthene | 6.4^a^ | 25^d^ | n.d.^e^ |
| Fluorene | 16.6^a^ | 104^d^ | n.d.^e^ |
| Phenanthrene | 67.3^a^ | 540^d^ | 11.5±3.4^e^ |
| Anthracene | 5.9^a^ | 114^d^ | 0.1±0.2^e^ |
| Fluoranthene | 48.4^a^ | 299^d^ | 11±3.2^e^ |
| Pyrene | 43^a^ | 303^d^ | 15.7±5.8^e^ |
| Benzo(a)anthracene | 23.6^a^ | 158^d^ | 3.8±2^e^ |
| Chrysene | 46.1^a^ | 302^d^ | 18.8±3.8^e^ |
| Benzo(b+k) fluoranthene | n.d.^a^ | 302^d^ | 35.3±9.6^e^ |
| Benzo(a)pyrene | 17.5^a^ | 105^d^ | n.d.^e^ |
| Dibenz(a,h)anthracene | n.d.^a^ | 212^d^ | n.d.^e^ |
| Indeno(1,2,3-c,d)pyrene | 24.1^a^ | 159^d^ | 9.2±5^e^ |
| Benzo(g,h,i)perylene | 26.7^a^ | 215^d^ | n.d.^e^ |
| Total 16 PAHs | 352^a^ | 3034^d^ | 143±33.2^e^ |
| PBDEs | μg/kg | | |
| Total mono-BDEs | 1.01^a^ | 3^f^ | n.a.^c^ |
| Total di-BDEs | 2.73^a^ | 3.74^f^ | n.a.^c^ |
| Total tri-BDEs | 2.51^a^ | 11.8^f^ | 21.965^c^ |
| Total tetra-BDEs | 8.41^a^ | 84.6^f^ | 207.161^c^ |
| Total penta-BDEs | 8.52^a^ | 60^f^ | 253.783^c^ |
| Total hexa-BDEs | 5.27^a^ | 23.3^f^ | 114.026^c^ |
| Total hepta-BDEs | 3.81^a^ | 27.8^f^ | 478.426^c^ |
| Total octa-BDEs | n.a.^a^ | n.a.^f^ | n.a.^c^ |
| Total nona-BDEs | n.a.^a^ | n.a.^f^ | 939.755^c^ |
| Deca-BDE (BDE-209) | n.a.^a^ | 8840^f^ | 5319.057^c^ |
| Total PBDEs | 32.3 (Total mono-hepta BDEs) ^a^ | 9054^f^ | 7349.32^c^ |
| PCBs | μg/kg | | |
| Total mono-PCBs | 2.39^a^ | n.d.^a^ |  |
| Total di-PCBs | 122^a^ | n.d.^a^ |  |
| Total tri-PCBs | 294^a^ | n.d.^a^ |  |
| Total tetra-PCBs | 258^a^ | 5.11^a^ |  |
| Total penta-PCBs | 43.9^a^ | 4.92^a^ |  |
| Total hexa-PCBs | 15.9^a^ | 4.04^a^ |  |
| Total hepta-PCBs | 5.2^a^ | 0.32^a^ |  |
| Total octa-PCBs | n.d.^a^ | n.d.^a^ |  |
| Total nona-PCBs | n.d.^a^ | n.d.^a^ |  |
| Deca-PCB (PCB-209) | n.d.^a^ | n.d.^a^ |  |
| Total PCBs | 743^a^ | 14.1^a^ |  |

PAHs, polycyclic aromatic hydrocarbons; PBDEs, polybrominated diphenyl ethers; PCB, polychlorinated biphenyls; n.a., not applicable; n.d., not detectable.

^a^ Leung, A., Cai, Z.W., and Wong, M.H. (2006). Environmental contamination from electronic waste recycling at Guiyu, southeast China. *J. Mater. Cycles Waste Manage.* 8, 21-33. doi: 10.1007/s10163-005-0141-6.

^b^ Guo, Y., Huang, C., Zhang, H., and Dong, Q. (2009). Heavy metal contamination from electronic waste recycling at Guiyu, southeastern China. *J. Environ. Qual.* 38, 1617-1626. doi: 10.2134/jeq2008.0398.

^c^ Nie, X., Fan, C., Wang, Z., Su, T., Liu, X., and An, T. (2015). Toxic assessment of the leachates of paddy soils and river sediments from e-waste dismantling sites to microalga, *Pseudokirchneriella subcapitata*. *Ecotox. Environ. Safe.* 111, 168-176. doi: 10.1016/j.ecoenv.2014.10.012.

^d^ Xu, P., Tao, B., Ye, Z., Zhao, H., Ren, Y., Zhang, T., et al. (2016). Polycyclic aromatic hydrocarbon concentrations, compositions, sources, and associated carcinogenic risks to humans in farmland soils and riverine sediments from Guiyu, China. *J. Environ. Sci.* 48, 102-111. doi: 10.1016/j.jes.2015.11.035.

^e^ Leung, A.O.W., Cheung, K.C., and Wong, M.H. (2015). Spatial distribution of polycyclic aromatic hydrocarbons in soil, sediment, and combusted residue at an e-waste processing site in southeast China. *Environ. Sci. Pollut. Res.* 22, 8786-8801. doi: 10.1007/s11356-013-1465-8.

^f^ Huang, Y., Zhang, D., Yang, Y., Zeng, X., and Ran, Y. (2018). Distribution and partitioning of polybrominated diphenyl ethers in sediments from the pearl river delta and Guiyu, south China. *Environ. Pollut.* 235, 104-112. doi: 10.1016/j.envpol.2017.12.049.
