# Supplementary material for: Adaptive Evolution of Sphingobium hydrophobicum C1T in Electronic Waste Contaminated River Sediment
Source: Front Microbiol. 2019 Oct 2;10:2263. doi: 10.3389/fmicb.2019.02263 (PMC6783567; doi:10.3389/fmicb.2019.02263)
Supplement: Supplementary file 1 [file Data_Sheet_1.zip › Data Sheet 1/Supplementary Materials/Table S2.docx]

**Table S2.** Isolation sources, main features, genome statistics and accession numbers of the 19 *Sphingobium* strains.

| Strain | Isolation source | Main Feature | Size (Mb) | GC% | Sequencing status  (chromosomes/plasmids) | Accession numbers |
| --- | --- | --- | --- | --- | --- | --- |
| *S. abikonense* NBRC16140 | An oil-soaked soil, Japan | Metabolizing dibenzothiophene to sulfur-containing organic acid compounds | 3.75 | 63.5 | Draft | NZ_BCYT01000001.1 - NZ_BCYT01000050.1 |
| *S. amiense* NBRC102518 | A river sediment sample obtained in Ami-machi, Ibaraki, Japan | Degrading nonylphenol | 4.54 | 64.7 | Draft | NZ_BCUN01000001.1 - NZ_BCUN01000130.1 |
| *S. baderi* LL03 | A hexachlorocyclohexane (HCH)-contaminated soil at Spolana Neratovice, Czech Republic | Degrading α-, γ- and δ- isomers of HCH | 4.85 | 63.6 | Draft | NZ_ATIB01000001.1 - NZ_ATIB01000092.1 |
| *S. barthaii* KK22 | A microbial consortium that grew on diesel fuel originally recovered from cattle pasture soil in the Gulf region of Texas, USA | Degrading high molecular weight polycyclic aromatic hydrocarbons | 4.92 | 64.7 | Draft | NZ_BATN01000001 - NZ_BATN01000252 |
| *S. chinhatense* IP26 | A highly HCH-contaminated soil, North India | Degrading α-, β-, γ- and δ-HCH isomers faster than *S. indicum* B90A | 5.85 | 64.1 | Draft | NZ_AUDA01000001.1 - NZ_AUDA01000236.1 |
| *S. chlorophenolicum* L-1 | A pentachlorophenol (PCP)-contaminated soil in Minnesota, America | Mineralizing PCP | 4.57 | 63.8 | Complete (2/1) | NC_015593.1 - NC_015595.1 |
| *S. chungbukense* DJ77 | Contaminated sediment of an industrial complex near Taejon, Republic of Korea | Degrading biphenyl, phenanthrenes and a variety of monocyclic aromatic hydrocarbons | 5.52 | 63.3 | Draft | NZ_LBIC01000001.1 - NZ_LBIC01000040.1 |
| *S. cloacae* NBRC 102517 | A sewage-treatment plant in Tokyo, Japan | Degrading nonylphenol | 4.29 | 64.6 | Draft | NZ_BCUM01000001.1 - NZ_BCUM01000169.1 |
| *S. czechense* LL01 | HCH-contaminated soil at Spolana Neratovice, Czech. | Degrading all four HCH isomers | 4.66 | 63.6 | Draft | NZ_KQ130434.1 - NZ_KQ130451.1 |
| *S. herbicidovorans* NBRC16415 | A soil column with (RS)-2-(2,4-dichlorophenoxy) propionic acid as the carbon and energy source, Austria | Degrading 2,4-DP, 2,4-D, 2,4-DB, MCPP | 4.03 | 62.4 | Draft | NZ_JFZA02000001.1 - NZ_JFZA02000062.1 |
| *S. indicum* B90A | The rice rhizosphere, Cuttack, India. | Degrading all isomers of HCH | 4.08 | 65.0 | Draft | NZ_AJXQ01000001.1 - NZ_AJXQ01000149.1 |
| *S. japonicum* UT26S | Upland fields in Japan where gamma-HCH had been applied for 15 years | Degradingα-, γ- and δ- isomers of HCH | 4.42 | 64.9 | Complete (2/3) | NC_014005.1 NC_014006.1  NC_014007.1  NC_014009.1  NC_014013.1 |
| *S. lactosutens* DS20 | An HCH dump site at Lucknow, India | Not degrading HCH | 5.36 | 63.0 | Draft | NZ_ATDP01000001.1 - NZ_ATDP01000110.1 |
| *S. lucknowense* F2 | An HCH dump site at Ummari village in Lucknow, India | Degrading all four HCH isomers | 4.44 | 64.3 | Draft | NZ_JANF02000001.1 - NZ_JANF02000103.1 |
| *S. quisquiliarum* P25 | An HCH dump site located in the northern part of India | Degrading all four HCH isomers | 4.17 | 64.0 | Draft | NZ_ATHO01000001.1 - NZ_ATHO01000181.1 |
| *S. ummariense* RL-3 | An HCH dump site located in the northern part of India | Degrading all four HCH isomers | 4.75 | 65.0 | Draft | NZ_AUWY01000001.1 - NZ_AUWY01000139.1 |
| *S. xenophagum* NBRC107872 | A water sample from river Elbe near Hamburg, Germany | Oxidizing a wide range of (substituted) naphthalenesulfonates to the corresponding (substituted) salicylates; reducing the azo bond of sulfonated azo dyes under anaerobic conditions | 4.49 | 62.9 | Draft | NZ_BARE01000001.1 - NZ_BARE01000138.1 |
| *S. xenophagum* QYY | Sludge samples from bromoamine acid producing workshop of Zhaoyuan Chemical Plant in Shandong Province of China | Degrading bromoamine acid | 4.22 | 63.1 | Draft | NZ_AKIB01000001.1 - NZ_AKIB01000129.1 |
| *S. yanoikuyae* ATCC51230 | Clinical specimen, Japan | Uncertainty | 5.53 | 64.3 | Draft | NZ_JH992904.1 - NZ_JH992912.1 |
