# Supplementary material for: Adaptive Evolution of Sphingobium hydrophobicum C1T in Electronic Waste Contaminated River Sediment
Source: Front Microbiol. 2019 Oct 2;10:2263. doi: 10.3389/fmicb.2019.02263 (PMC6783567; doi:10.3389/fmicb.2019.02263)
Supplement: Supplementary file 1 [file Data_Sheet_1.zip › Data Sheet 1/Supplementary Materials/Table S3.docx]

**Table S3.** ANI values based on Blast and percentage of aligned sequence (Coverage) in comparison with the C1^T^ genome.

|  | ANI [Coverage] |  | ANI [Coverage] |
| --- | --- | --- | --- |
| *S. abikonense* NBRC16140 | 79.1 [50.41] | *S. indicum* B90A | 78.01 [43.59] |
| *S. amiense* NBRC102518 | 78.81 [48.95] | *S. japonicum* UT26S | 78.2 [44.97] |
| *S. baderi* LL03 | 77.7 [44.14] | *S. lactosutens* DS20 | 78.15 [51.18] |
| *S. barthaii* KK22 | 77.75 [46.32] | *S. lucknowense* F2 | 77.86 [37.58] |
| *S. chinhatense* IP26 | 78.3 [48.12] | *S. quisquiliarum* P25 | 77.73 [43.16] |
| *S. chlorophenolicum* L-1 | 77.54 [43.19] | *S. ummariense* RL-3 | 78.33 [48.84] |
| *S. chungbukense* DJ77 | 77.61 [49.4] | *S. xenophagum* NBRC 107872 | 95.27 [74.55] |
| *S. cloacae* NBRC102517 | 77.96 [43.79] | *S. xenophagum* QYY | 97.69 [78.93] |
| *S. czechense* LL01 | 80.48 [55.51] | *S. yanoikuyae* ATCC51230 | 79.32 [50.58] |
| *S. herbicidovorans* NBRC 16415 | 76.44 [43.96] |  |  |
