# Supplementary material for: Adaptive Evolution of Sphingobium hydrophobicum C1T in Electronic Waste Contaminated River Sediment
Source: Front Microbiol. 2019 Oct 2;10:2263. doi: 10.3389/fmicb.2019.02263 (PMC6783567; doi:10.3389/fmicb.2019.02263)
Supplement: Supplementary file 1 [file Data_Sheet_1.zip › Data Sheet 1/Supplementary Materials/Table S4.docx]

**Table S4.** Number of the orthologs between strain C1^T^ and *S. xenophagum* strains under different selection pressures.

|  | Orthologs | No substitution | Positive selection | Negative selection | Neutral evolution |
| --- | --- | --- | --- | --- | --- |
| C1^T^-QYY | 3173 | 2673 | 201 | 225 | 74 |
| C1^T^-NBRC107872 | 2973 | 179 | 25 | 2701 | 68 |
