# Supplementary material for: Adaptive Evolution of Sphingobium hydrophobicum C1T in Electronic Waste Contaminated River Sediment
Source: Front Microbiol. 2019 Oct 2;10:2263. doi: 10.3389/fmicb.2019.02263 (PMC6783567; doi:10.3389/fmicb.2019.02263)
Supplement: Supplementary file 1 [file Data_Sheet_1.zip › Data Sheet 1/Supplementary Materials/Table S5.docx]

**Table S5.** List of oxygenase genes, multidrug efflux pump genes, heavy metal resistance genes and their Ka/Ks values.

| Gene | Location | Annotation | Ka/Ks | P-Value(Fisher) | |
| --- | --- | --- | --- | --- | --- |
| Oxygenase |  |  |  |  | |
| Chromosome1_00870 | Chromosome1_854334-855710 | Probable cytochrome P450 | 0.001 | n.a. | |
| Chromosome1_01298 | Chromosome1_1303245-1304477 | Camphor 5-monooxygenase | N.A. | N.A. | |
| Chromosome1_01339 | Chromosome1_1343055-1343981 | Catechol 2,3-dioxygenase | N.A. | N.A. | |
| Chromosome1_01510 | Chromosome1_1511165-1512052 | Phe-4-monooxygenase | 0.001 | n.a. | |
| Chromosome1_01511 | Chromosome1_1512049-1513089 | 4-hydroxyphenylpyruvate dioxygenase | 0.001 | n.a. | |
| Chromosome1_01512 | Chromosome1_1513117-1514388 | Homogentisate 1,2-dioxygenase | 50 | 0 | |
| Chromosome2_00006 | Chromosome2_3084-3998 | Alpha-ketoglutarate-dependent 2,4-dichlorophenoxyacetate dioxygenase | n.a. | n.a. | |
| Chromosome2_00032 | Chromosome2_31595-32821 | 3-hydroxy-9,10-secoandrosta-1,3,5(10)-triene-9,17-dione monooxygenase | 0.001 | 0 | |
| Chromosome2_00072 | Chromosome2_84173-85405 | 6-hydroxynicotinate 3-monooxygenase | n.a. | n.a. | |
| Chromosome2_00088 | Chromosome2_101245-102465 | Camphor 5-monooxygenase | 0.001 | 0 | |
| Chromosome2_00143 | Chromosome2_160031-160987 | Catechol 2,3-dioxygenase II | n.a. | n.a. | |
| Chromosome2_00163 | Chromosome2_175923-177308 | Biphenyl dioxygenase subunit alpha | n.a. | n.a. | |
| Chromosome2_00164 | Chromosome2_177337-177912 | Benzene 1,2-dioxygenase subunit beta | n.a. | n.a. | |
| Chromosome2_00172 | Chromosome2_184018-185241 | Camphor 5-monooxygenase | 50 | 0 | |
| Chromosome2_00185 | Chromosome2_196443-197657 | Toluate 1,2-dioxygenase subunit alpha | 0.001 | n.a. | |
| Chromosome2_00288 | Chromosome2_302300-303784 | Lignostilbene-alpha,beta-dioxygenase isozyme I | 0.001 | n.a. | |
| Chromosome2_00401 | Chromosome2_424728-425183 | Protocatechuate 4,5-dioxygenase alpha chain | n.a. | n.a. | |
| Chromosome2_00402 | Chromosome2_425183-426079 | Protocatechuate 4,5-dioxygenase beta chain | 0.001 | n.a. | |
| Chromosome2_00408 | Chromosome2_430407-431576 | 4-hydroxybenzoate 3-monooxygenase | n.a. | n.a. | |
| Chromosome2_00589 | Chromosome2_636798-638267 | Lignostilbene-alpha,beta-dioxygenase isozyme III | 0.001 | n.a. | |
| Plasmid2_00010 | Plasmid2_6782-8110 | Phthalate 4,5-dioxygenase oxygenase subunit | N.A. | N.A. | |
| Plasmid2_00023 | Plasmid2_20100-21197 | Naphthalene 1,2-dioxygenase subunit alpha | 0.001 | n.a. | |
| Plasmid2_00042 | Plasmid2_37863-38276 | Protocatechuate 4,5-dioxygenase alpha chain | n.a. | n.a. | |
| Plasmid2_00043 | Plasmid2_38276-39139 | Protocatechuate 4,5-dioxygenase beta chain | 0.001 | n.a. | |
| Plasmid2_00056 | Plasmid2_51030-52364 | Phthalate 4,5-dioxygenase oxygenase subunit | 0.001 | n.a. | |
| Plasmid2_00231 | Plasmid2_210189-211556 | Biphenyl dioxygenase subunit alpha | N.A. | N.A. | |
| Plasmid2_00232 | Plasmid2_211602-211772 | Biphenyl dioxygenase subunit beta | N.A. | N.A. | |
| multidrug efflux pump |  |  |  |  | |
| Chromosome1_00123 | Chromosome1_109236-110636 | *oprM*; outer membrane protein, multidrug efflux system | n.a. | n.a. | |
| Chromosome1_00124 | Chromosome1_110640-113792 | *acrB*; multidrug efflux pump | n.a. | n.a. | |
| Chromosome1_00125 | Chromosome1_113796-114944 | *acrA*; membrane fusion protein, multidrug efflux system | n.a. | n.a. | |
| Chromosome1_01576 | Chromosome1_1580615-1582144 | Multidrug resistance outer membrane protein MdtP precursor | n.a. | n.a. | |
| Chromosome1_01577 | Chromosome1_1582137-1583306 | *emrA*; membrane fusion protein, multidrug efflux system | n.a. | n.a. | |
| Chromosome1_01578 | Chromosome1_1583318-1584850 | *emrB*; MFS transporter, DHA2 family, multidrug resistance protein | 0.001 | n.a. | |
| Chromosome1_02069 | Chromosome1_2056307-2057350 | *acrA*; membrane fusion protein, multidrug efflux system | n.a. | n.a. | |
| Chromosome1_02070 | Chromosome1_2057360-2060464 | *acrB*; multidrug efflux pump | 0.001 | 0 | |
| Chromosome2_00600 | Chromosome2_649531-650658 | *acrA*; membrane fusion protein, multidrug efflux system | n.a. | n.a. | |
| Chromosome2_00601 | Chromosome2_650669-653818 | *acrB*; multidrug efflux pump | 0.001 | 0 | |
| Chromosome2_00602 | Chromosome2_653815-655170 | *oprM*; outer membrane protein, multidrug efflux system | 0.001 | 0 | |
| heavy metal resistance |  |  |  |  | |
| Chromosome1_01100 | Chromosome1_1094502-1095914 | *chrA*; chromate transporter | N.A. | N.A. |  |
| Chromosome1_01103 | Chromosome1_1097228-1098508 | *czcC*; outer membrane protein, cobalt-zinc-cadmium efflux system | N.A. | N.A. |  |
| Chromosome1_01104 | Chromosome1_1098508-1099755 | *czcB*; membrane fusion protein, cobalt-zinc-cadmium efflux system | N.A. | N.A. |  |
| Chromosome1_01105 | Chromosome1_1099745-1102936 | *czcA*; cobalt-zinc-cadmium resistance protein CzcA | N.A. | N.A. |  |
| Chromosome1_01126 | Chromosome1_1125343-1126104 | *arsH*; arsenical resistance protein ArsH | N.A. | N.A. |  |
| Chromosome1_01127 | Chromosome1_1126107-1127174 | *TC.ACR3*; arsenite transporter, ACR3 family | N.A. | N.A. |  |
| Chromosome1_01128 | Chromosome1_1127190-1127621 | *ARSC1*; arsenate reductase | N.A. | N.A. |  |
| Chromosome1_01387 | Chromosome1_1390225-1391256 | *TC.ACR3*; arsenite transporter, ACR3 family | 0.350555 | 0 |  |
| Chromosome1_01388 | Chromosome1_1391256-1391666 | *ARSC1*; arsenate reductase | n.a. | n.a. |  |
| Chromosome1_01843 | Chromosome1_1842033-1842377 | *ARSC1*; arsenate reductase | 50 | 0 |  |
| Chromosome1_02601 | Chromosome1_2614252-2615448 | *chrA*; chromate transporter | 0.308093 | 0.051695 |  |
| Chromosome2_00283 | Chromosome2_293358-296516 | *czcA*; cobalt-zinc-cadmium resistance protein CzcA | 0.001 | 0 |  |
| Chromosome2_00284 | Chromosome2_296506-297750 | *czcB*; membrane fusion protein, cobalt-zinc-cadmium efflux system | 50 | 0 |  |
| Chromosome2_00285 | Chromosome2_297750-299018 | *czcC*; outer membrane protein, cobalt-zinc-cadmium efflux system | n.a. | n.a. |  |
| Chromosome2_00373 | Chromosome2_395551-396468 | *pcoB*; copper resistance protein B | n.a. | n.a. |  |
| Chromosome2_00378 | Chromosome2_399625-400005 | *copC*; copper resistance protein C | n.a. | n.a. |  |
| Chromosome2_00379 | Chromosome2_400036-400872 | *pcoD*; copper resistance protein D | 50 | 0 |  |
| Plasmid1_00229 | Plasmid1_218071-219486 | *chrA*; chromate transporter | N.A. | N.A. |  |
| Plasmid2_00192 | Plasmid2_161951-163366 | *chrA*; chromate transporter | N.A. | N.A. |  |
| Plasmid2_00243 | Plasmid2_222421-223797 | *merA*; mercuric reductase | N.A. | N.A. |  |
| Plasmid2_00244 | Plasmid2_223877-224206 | *merP*; periplasmic mercuric ion binding protein | N.A. | N.A. |  |
| Plasmid2_00245 | Plasmid2_224219-224614 | *merT*; mercuric ion transport protein | N.A. | N.A. |  |
| Plasmid2_00249 | Plasmid2_226102-226518 | *merT*; mercuric ion transport protein | N.A. | N.A. |  |
| Plasmid4_00008 | Plasmid4_5343-5714 | *copC*; copper resistance protein C | N.A. | N.A. |  |
| Plasmid4_00009 | Plasmid4_5716-6645 | *pcoD*; copper resistance protein D | N.A. | N.A. |  |
| Plasmid4_00011 | Plasmid4_7227-7655 | *ARSC1*; arsenate reductase | N.A. | N.A. |  |
| Plasmid4_00012 | Plasmid4_7655-8941 | *arsB*; arsenical pump membrane protein | N.A. | N.A. |  |
| Plasmid4_00013 | Plasmid4_8941-9294 | *arsH*; arsenical resistance protein ArsH | N.A. | N.A. |  |
| Plasmid4_00014 | Plasmid4_9291-9686 | *arsH*; arsenical resistance protein ArsH | N.A. | N.A. |  |
| Plasmid4_00034 | Plasmid4_26767-28044 | *czcC*; outer membrane protein, cobalt-zinc-cadmium efflux system | 0.045132 | 7.8E-48 |  |
| Plasmid4_00035 | Plasmid4_28044-29219 | *czcB*; membrane fusion protein, cobalt-zinc-cadmium efflux system | 0.023278 | 2.19E-77 |  |
| Plasmid4_00036 | Plasmid4_29223-31913 | *czcA*; cobalt-zinc-cadmium resistance protein CzcA | 0.083382 | 1.4E-137 |  |
| Plasmid4_00037 | Plasmid4_31949-32467 | *czcA*; cobalt-zinc-cadmium resistance protein CzcA | N.A. | N.A. |  |
| Plasmid4_00095 | Plasmid4_76009-77220 | *pcoB*; copper resistance protein B | N.A. | N.A. |  |

Ka/Ks and P-Value were calculated based on orthologs between strains C1^T^ and QYY.

Positive selection: Ka/Ks > 1, P-value (Fisher) < 0.05.

Negative selection: Ka/Ks < 1, P-value (Fisher) < 0.05.

Neutral evolution: P-value (Fisher) ≥ 0.05.

N.A., not applicable due to no corresponding ortholog in strain QYY.

n.a., not applicable due to no synonymous and non-synonymous substitution between the orthologs.
