# Supplementary material for: Adaptive Evolution of Sphingobium hydrophobicum C1T in Electronic Waste Contaminated River Sediment
Source: Front Microbiol. 2019 Oct 2;10:2263. doi: 10.3389/fmicb.2019.02263 (PMC6783567; doi:10.3389/fmicb.2019.02263)
Supplement: Supplementary file 1 [file Data_Sheet_1.zip › Data Sheet 1/Supplementary Materials/Table S6.docx]

**Table S6.** Information of two-component regulatory systems in strain C1^T^.

| Two-component regulatory systems | Function | Location | State |
| --- | --- | --- | --- |
| PhoR-PhoB | Phosphate starvation response | Chromosome 1 | Complete |
| AlgZ-AlgR | Alginate production | Chromosome 1 | AlgZ missing |
| GlnL-GlnG | Nitrogen regulation | Chromosome 1 | Complete |
| NtrY-NtrX | Nitrogen regulation | Chromosome 1 | Complete |
| CheA-CheYBV | Chemotaxis | Chromosome 1 | CheV missing |
| CckA-CtrA/CpdR | Cell cycle control | Chromosome 1 | Complete |
| FlrB-FlrC | Polar flagellar synthesis | Chromosome 1 | Complete |
| ChvG-ChvI | Acidity sensing | Chromosome 1 | Complete |
| RegB-RegA | Redox response | Chromosome 1 | Complete |
| FixL-FixJ | Nitrogen fixation and/or microaerobic respiration | Chromosome 1, Plasmid 1 and Plasmid 3 (3 sets) | Complete |
| DctB-DctD | C4-dicarboxylate transport | Chromosome 2 | Complete |
