# Supplementary figures and images for: Adaptive Evolution of Sphingobium hydrophobicum C1T in Electronic Waste Contaminated River Sediment
Source: Front Microbiol. 2019 Oct 2;10:2263. doi: 10.3389/fmicb.2019.02263 (PMC6783567; doi:10.3389/fmicb.2019.02263)

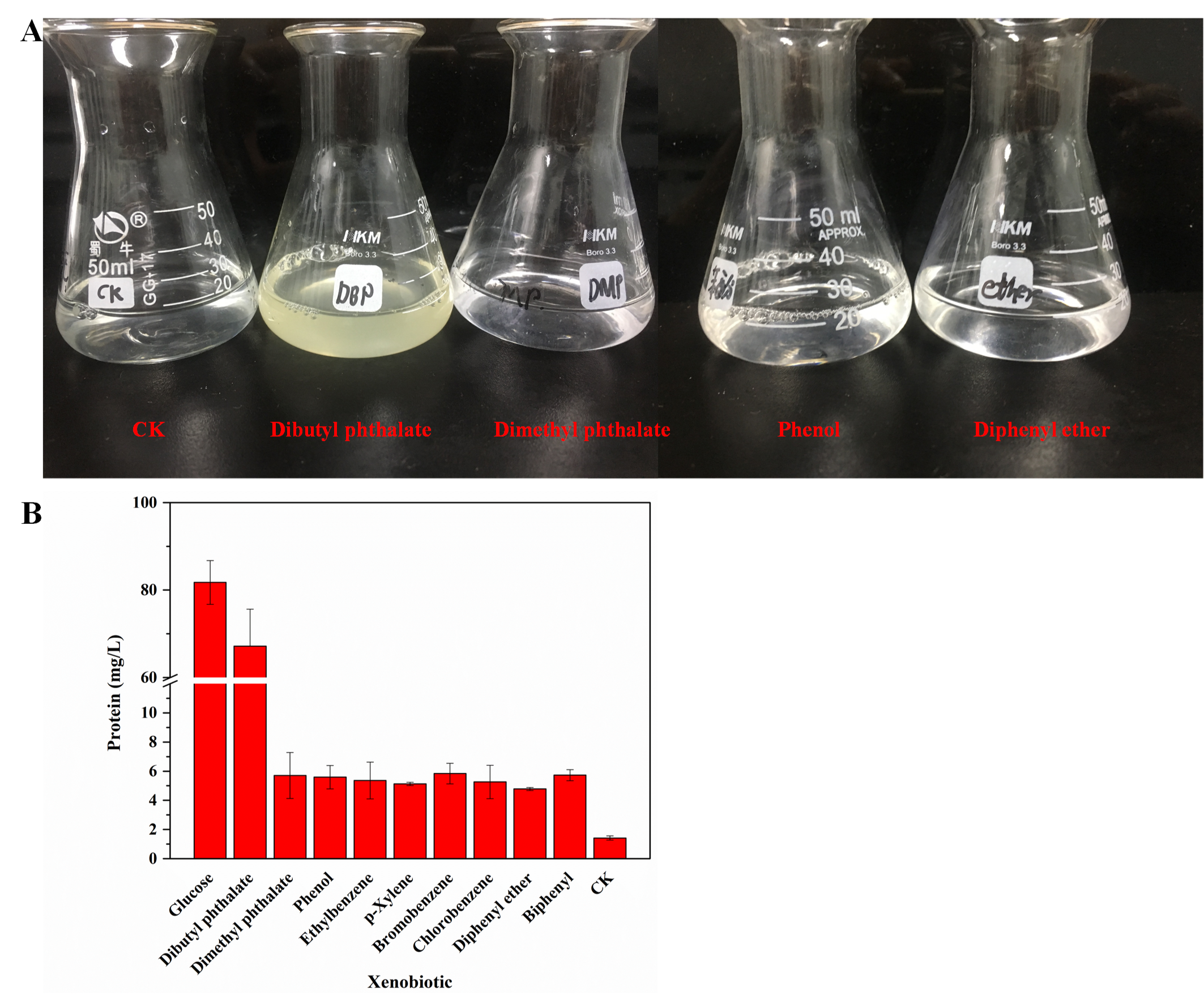

Supplement: Supplementary file 1 [file Data_Sheet_1.zip › Data Sheet 1/Supplementary Materials/Figure S1.tif]

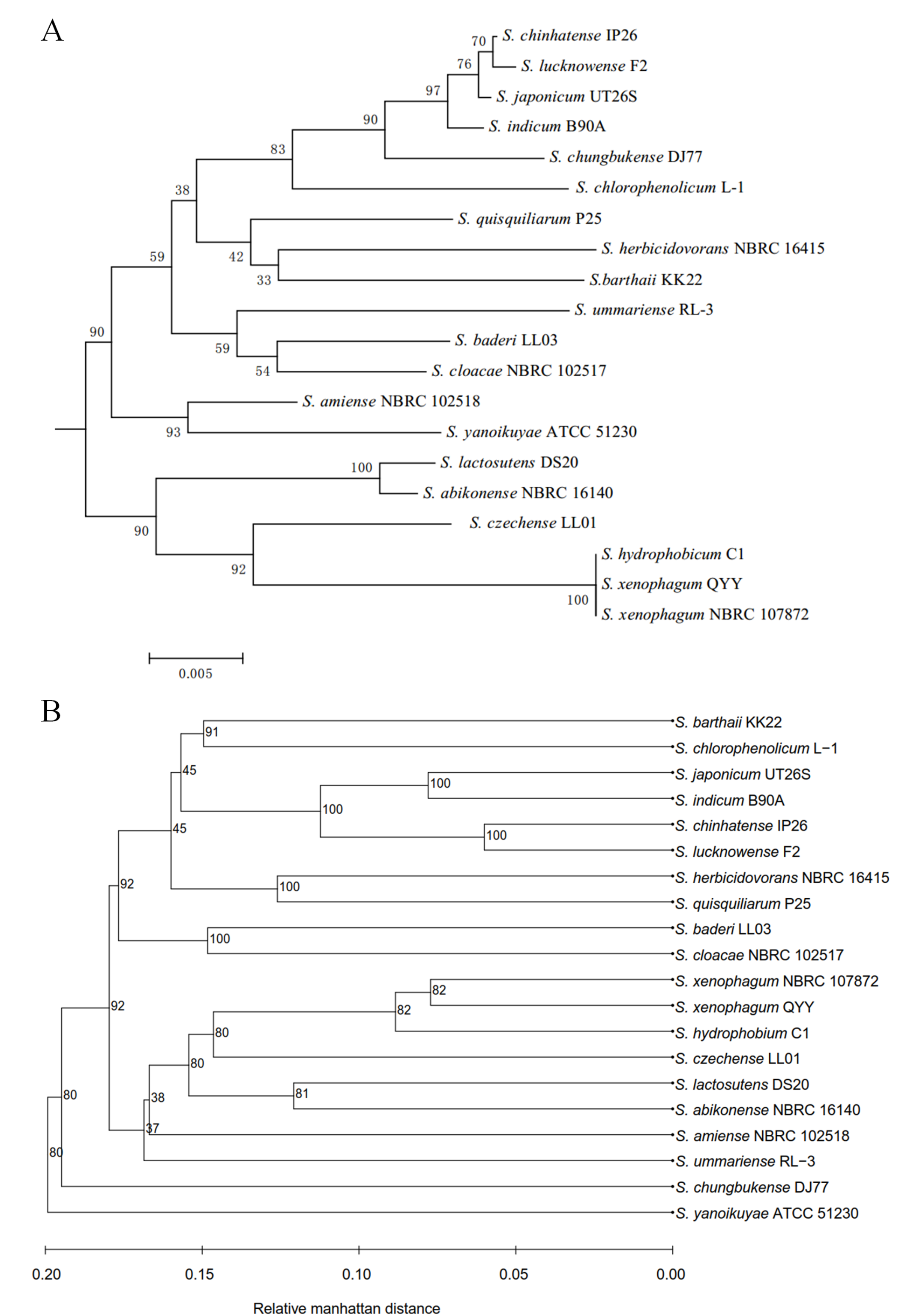

Supplement: Supplementary file 1 [file Data_Sheet_1.zip › Data Sheet 1/Supplementary Materials/Figure S2.tif]

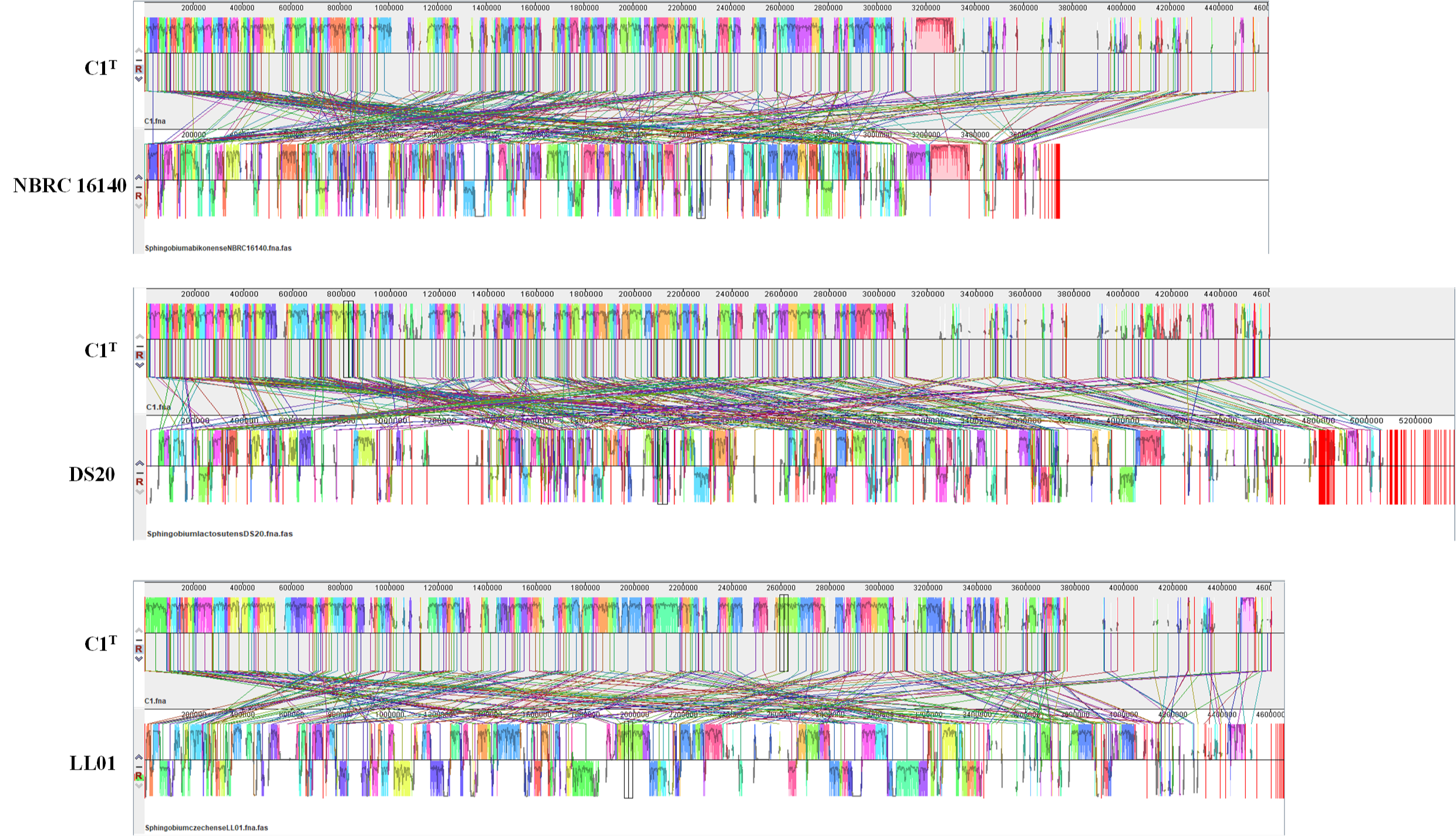

Supplement: Supplementary file 1 [file Data_Sheet_1.zip › Data Sheet 1/Supplementary Materials/Figure S3.tif]

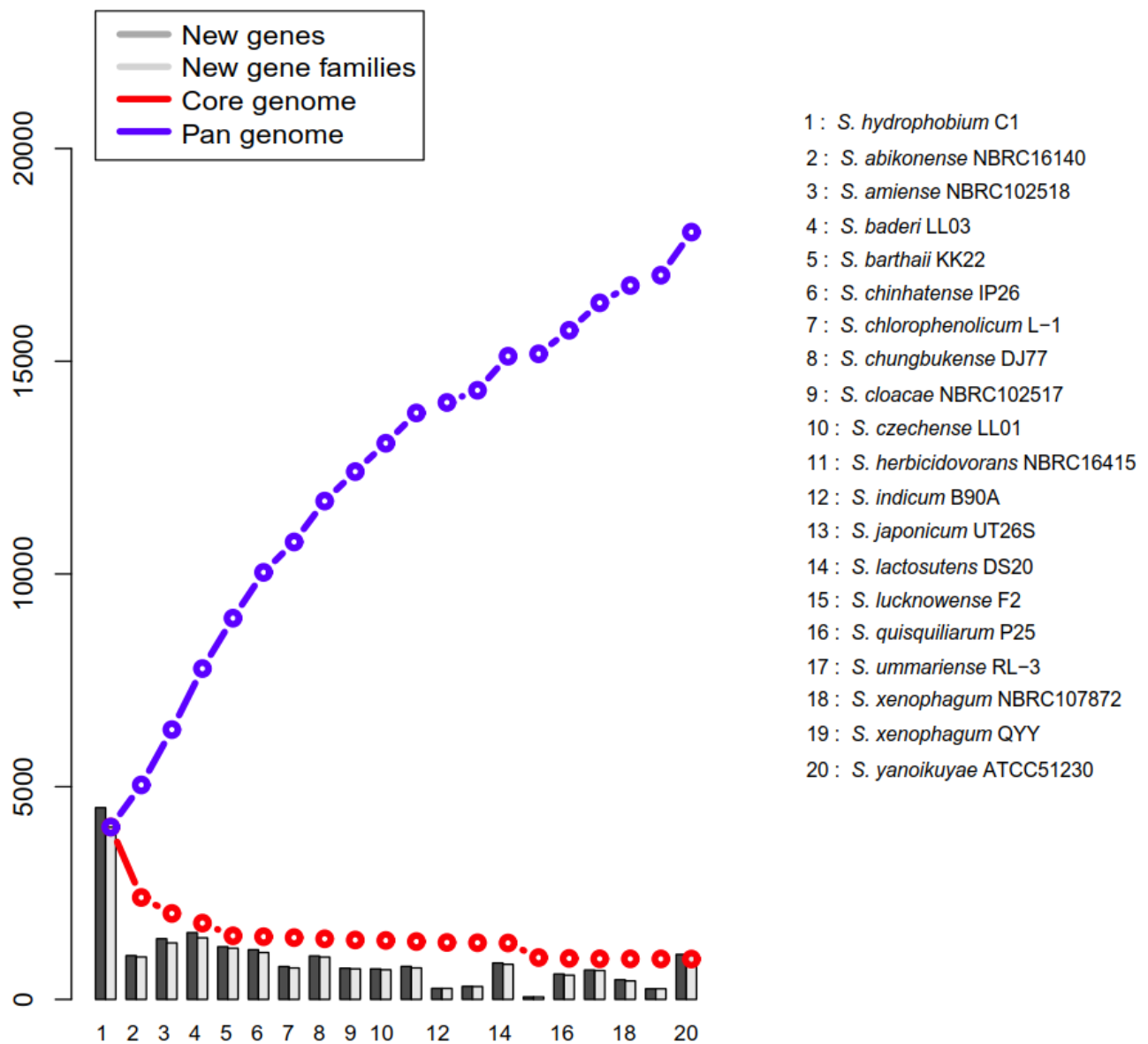

Supplement: Supplementary file 1 [file Data_Sheet_1.zip › Data Sheet 1/Supplementary Materials/Figure S4.tif]

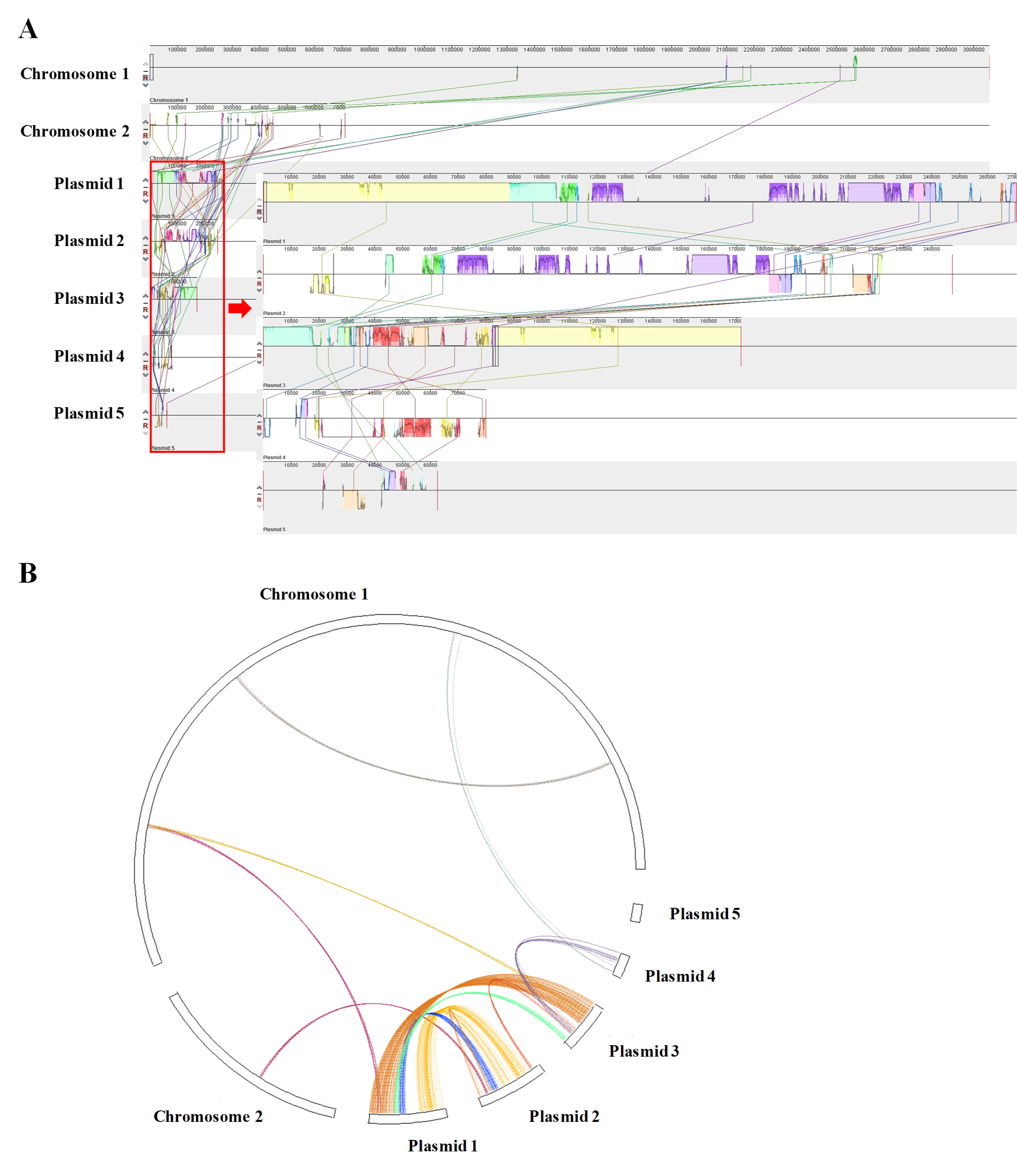

Supplement: Supplementary file 1 [file Data_Sheet_1.zip › Data Sheet 1/Supplementary Materials/Figure S5.tif]

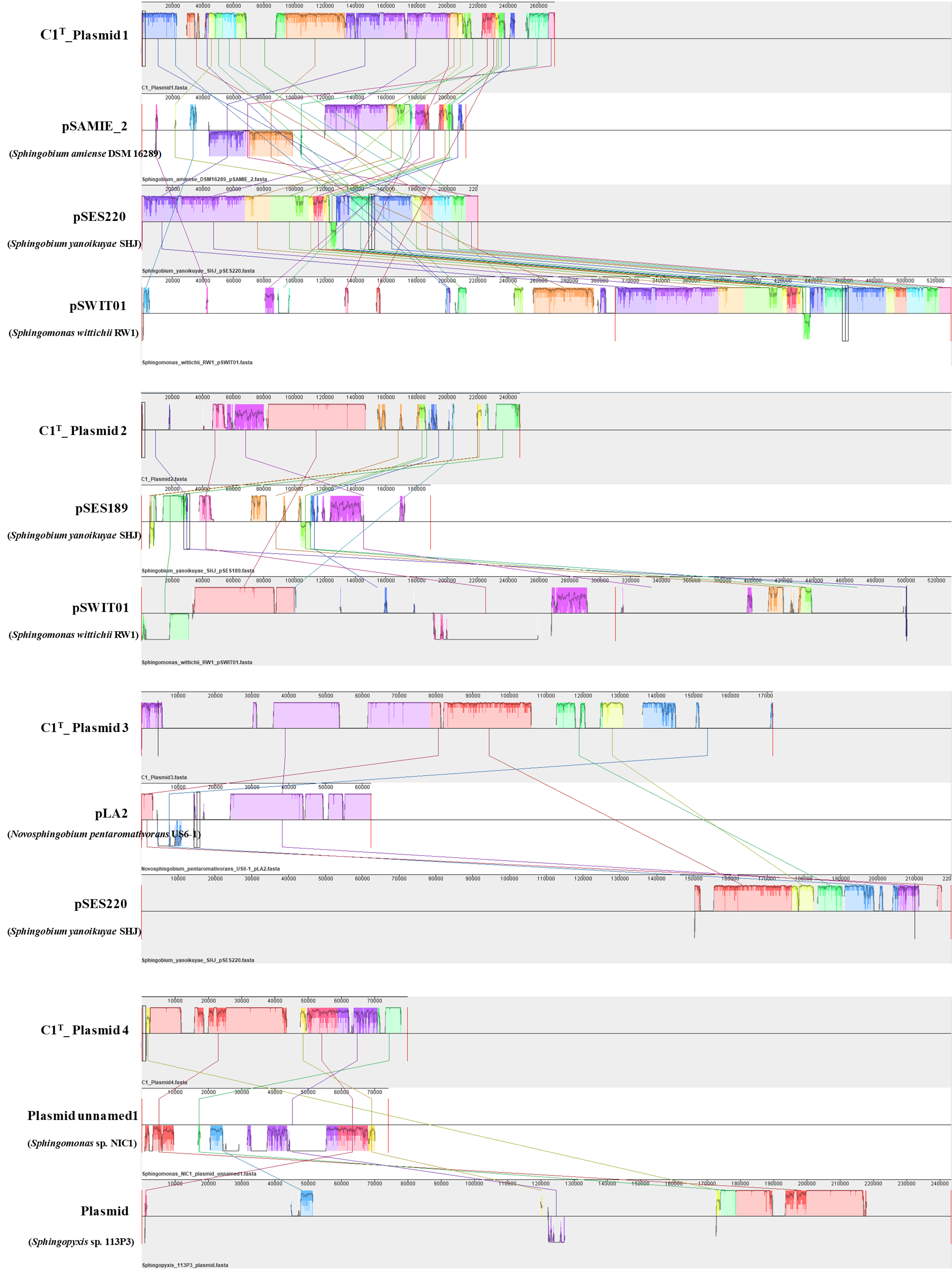

Supplement: Supplementary file 1 [file Data_Sheet_1.zip › Data Sheet 1/Supplementary Materials/Figure S7.tif]

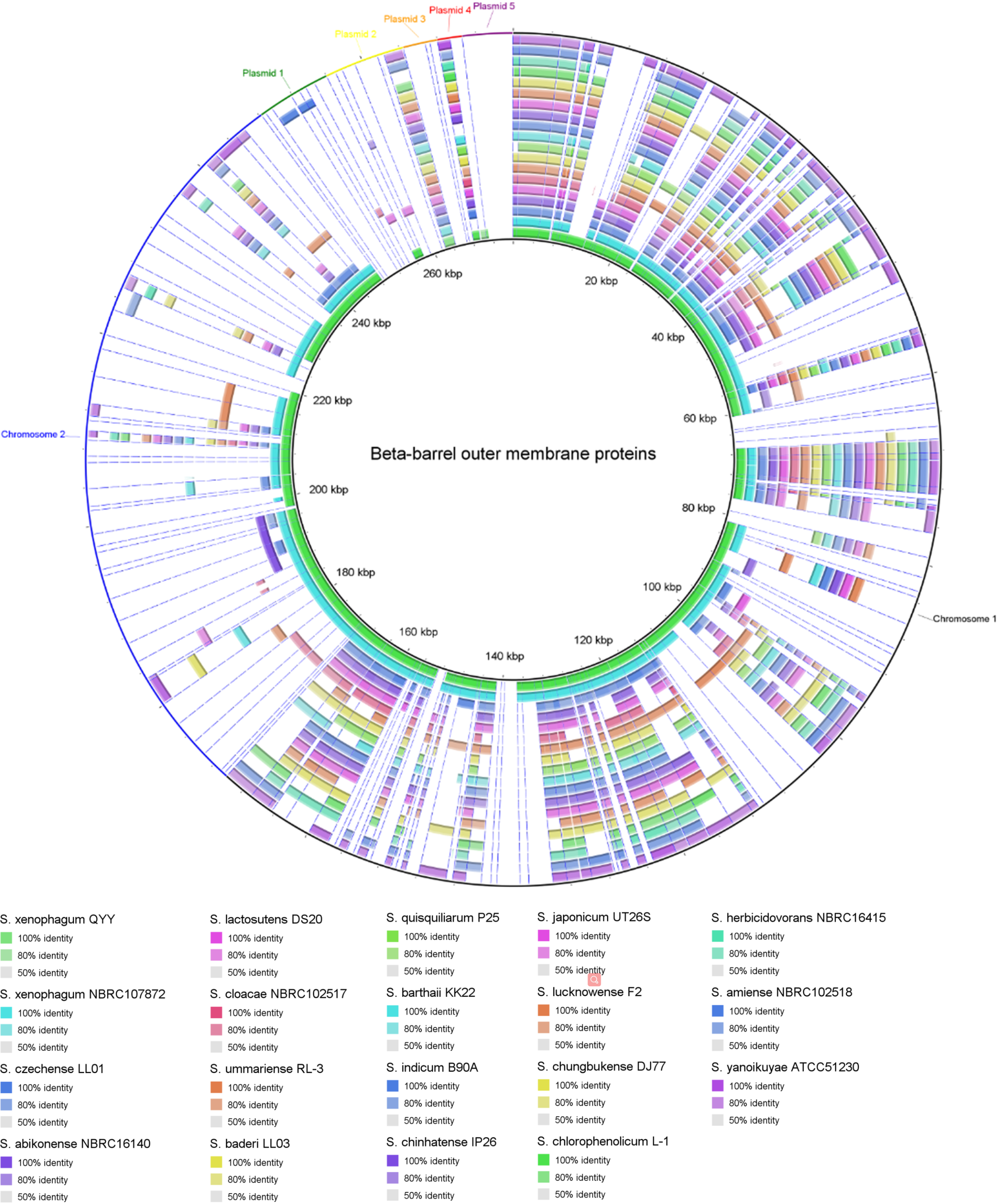

Supplement: Supplementary file 1 [file Data_Sheet_1.zip › Data Sheet 1/Supplementary Materials/Figure S8.tif]

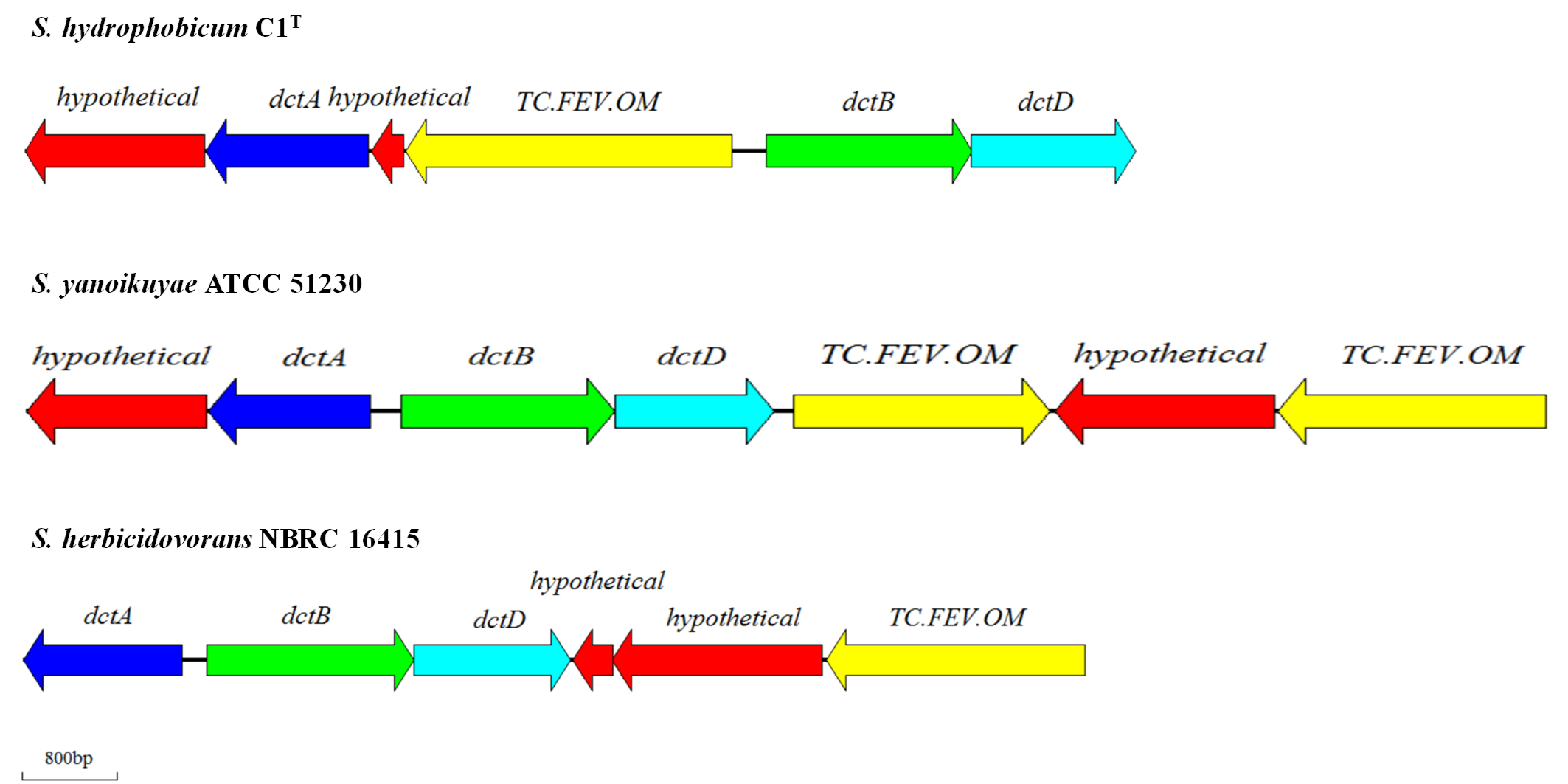

Supplement: Supplementary file 1 [file Data_Sheet_1.zip › Data Sheet 1/Supplementary Materials/Figure S9.tif]
